# High‐Yield Bioproduction of Extracellular Vesicles from Stem Cell Spheroids via Millifluidic Vortex Transport

**DOI:** 10.1002/adma.202412498

**Published:** 2024-11-12

**Authors:** Elliot Thouvenot, Laura Charnay, Noa Burshtein, Jean‐Michel Guigner, Léonie Dec, Damarys Loew, Amanda K.A. Silva, Anke Lindner, Claire Wilhelm

**Affiliations:** ^1^ Laboratoire Physique des Cellules et Cancer PCC CNRS UMR168 Institut Curie Sorbonne Université, PSL Research University Paris 75005 France; ^2^ Laboratoire de Physique et Mécanique des Milieux Hétérogènes PMMH CNRS UMR7636 ESPCI Paris PSL Research University Sorbonne Université Université Paris Cité Paris 75005 France; ^3^ Institut de Minéralogie de Physique des Matériaux et de Cosmochimie (IMPMC) UMR CNRS 7590 MNHN IRD UR 206 Campus Jussieu Sorbonne Université Case courrier 115, 4 Place Jussieu, 75252 Paris Cedex 05 France; ^4^ Institut Curie CurieCoreTech Mass Spectrometry Proteomics PSL Research University Paris France; ^5^ Laboratoire Matière et Systèmes Complexes MSC, CNRS UMR7057, Université Paris Cité Paris 75006 France

**Keywords:** bioproduction, cross‐slot chip, extracellular vesicles, stem cell spheroids, vortex dynamics

## Abstract

Extracellular vesicles (EVs) are emerging as novel therapeutics, particularly in cancer and degenerative diseases. Nevertheless, from both market and clinical viewpoints, high‐yield production methods using minimal cell materials are still needed. Herein, a millifluidic cross‐slot chip is proposed to induce high‐yield release of biologically active EVs from less than three million cells. Depending on the flow rate, a single vortex forms in the outlet channels, exposing transported cellular material to high viscous stresses. Importantly, the chip accommodates producer cells within their physiological environment, such as human mesenchymal stem cells (hMSCs) spheroids, while facilitating their visualization and individual tracking within the vortex. This precise control of viscous stresses at the spheroid level allows for the release of up to 30000 EVs per cell at a Reynolds number of ≈400, without compromising cellular integrity. Additionally, it reveals a threshold initiating EV production, providing evidence for a stress‐dependent mechanism governing vesicle secretion. EVs mass‐produced at high Reynolds displayed pro‐angiogenic and wound healing capabilities, as confirmed by proteomic and cytometric analysis of their cargo. These distinct molecular signatures of these EVs, compared to those derived from monolayers, underscore the critical roles of the production method and the 3D cellular environment in EV generation.

## Graphical abstract

Stem cell spheroids, produced via a high‐throughput microfabrication technique, are transported within a vortex generated at the outlet channels of a cross‐slot millifluidic chip. For Reynolds number exceeding 170–200, intermittent high shear stresses applied to each spheroid within the vortex trigger a massive release of biologically active extracellular vesicles.

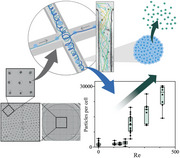

## 1 Introduction

Extracellular vesicles (EVs) are emerging as pivotal vectors of nanometric size delimited by biological membranes, now recognized as the major players in the far‐reaching cell‐to‐cell communication system. EVs are secreted by all types of cells and are present in all biological fluids,^[^ ^1^ ^]^ facilitating the exchange of complex cargo, including proteins, metabolites, and nucleic acids.^[^ ^1^, ^2^, ^3^ ^]^ Their biogenesis pathways are intricately intertwined with many physiological and pathological processes that govern cellular behavior, making them key regulators of essential cellular functions. Conversely, they also play significant roles in both the initiation and progression of diseases. Overall, EVs have demonstrated their involvement in modulating immune responses,^[^ ^4^, ^5^ ^]^ influencing tissue repair and regeneration,^[^ ^6^, ^7^, ^8^ ^]^ facilitating neo‐angiogenesis,^[^ ^9^, ^10^ ^]^ limiting organ fibrosis,^[^ ^11^, ^12^ ^]^ as well as favoring cancer progression and metastasis,^[^ ^13^, ^14^ ^]^ neurodegenerative diseases^[^ ^15^, ^16^ ^]^ or cardiovascular pathologies.^[^ ^17^, ^18^ ^]^

The ability of EVs to mirror the functions of their producer cells^[^ ^19^, ^20^ ^]^ along with their capacity to be engineered to enhance their therapeutic potential,^[^ ^21^, ^22^ ^]^ has positioned them as promising candidates for both cell‐free therapies, offering an alternative to conventional cell‐based approaches,^[^ ^23^ ^]^ and non‐invasive biomarker sources.^[^ ^24^ ^]^ As a consequence, research on EVs has exponentially increased in the last decade,^[^ ^25^ ^]^ with 116 clinical trials registered in 2022.^[^ ^26^ ^]^ However, a significant hurdle in realizing the full therapeutic potential of EVs lies in the challenge of producing them in therapeutic quantities and ensuring clinically‐compatible quality. This necessitates advancements in our understanding and control on EV release,^[^ ^27^ ^]^ as well as the development of innovative and performant bioprocesses.^[^ ^28^ ^]^ Current and widely used production methods take advantage of EVs release induced by starvation,^[^ ^29^, ^30^ ^]^ pH,^[^ ^31^, ^32^ ^]^ hypoxia,^[^ ^33^, ^34^ ^]^ or chemical activation.^[^ ^35^, ^36^, ^37^ ^]^ Yet, they yield low quantities of EVs per producer cell. Scaling out the culture systems, using hyperflasks, hollow fibers bioreactors, or cell culture on microcarriers in stirred tank bioreactors, enabled operators to increase the quantity of EVs by escalating the number of producer cells, although still requiring large quantity of cellular material to operate, without feasible control over the production process.^[^ ^38^, ^39^, ^40^ ^]^

To meet the need for producing EVs in sufficient quantities from a limited number of producer cells for clinical application, it remains thus imperative to enhance the yield per produced cell. In response to these demands, hydrodynamic‐based approaches have been proposed to augment EVs production per cell. The rational certainly lies in the acknowledged paradigm that mechanical forces serve as key modulators of cellular behavior, influencing processes ranging from cell differentiation^[^ ^21^ ^]^ to tissue development,^[^ ^41^ ^]^ tissue repair,^[^ ^42^ ^]^ modulation of the immune system^[^ ^43^ ^]^ or even cell death.^[^ ^44^ ^]^ Expanding upon these foundations, mechanical or viscous stresses have naturally been investigated as stimuli for EV release,^[^ ^45^ ^]^ indeed showcasing some enhancement in vesicle production yield.^[^ ^46^, ^47^, ^48^, ^49^ ^]^ However, existing techniques, such as shear‐stress‐induced production in high‐speed stirred tank^[^ ^50^ ^]^ exhibit notable drawbacks. While achieving high yields of EVs per cell, these techniques of EVs production in bioreactors are still limited by their dependence on a substantial quantity of producer cells, the lower biological relevance of cells cultured on beads compared to 3D configurations^[^ ^51^ ^]^ and their inability to precisely control the stress applied to the cellular material. Consequently, there is still an unmet need for methodologies that not only enhance the yield and adaptability of EV production, but also enable the control of the hydrodynamic conditions. This is crucial for understanding the intricate biological processes connecting mechanical cues and EV release.

Recent hydrodynamic‐based techniques successfully used for EV production rely on turbulent flow states, characterized by vortices of different sizes and strengths. Consequently, we propose the use of a cross‐slot chip that enables the formation of a single vortex, with properties directly controlled via the geometry and the applied Reynolds number. For the first time we use here a well‐controlled flow geometry outside the turbulent regime. A typical cross slot chip consists of two opposing inlets and two opposing outlets, where a Burger‐like vortex emerges above a critical Reynolds number. The resulting flow patterns have been extensively characterized in the past, using experiments and numerical simulations,^[^ ^52^, ^53^, ^54^, ^55^, ^56^, ^57^ ^]^ and transitions from stationary to time dependent flows or the appearance of secondary vortices have been observed. However, applications of this chip for biological or therapeutic applications remain scarce; primarily focusing on the characterization of mechanical properties of red blood or oral cancer cells in low Reynolds regimes,^[^ ^58^, ^59^, ^60^, ^61^, ^62^, ^63^ ^]^ or more recently, the transmembrane transport of nanomaterials at higher Reynolds.^[^ ^64^ ^]^ Here, we propose the use of a millifluidic cross slot chip to trigger EV production, leveraging direct control of vortex strength and vortical flow patterns. Additionally, biological material transported in the vortical flow can be visualized directly under the microscope, allowing to quantify the effect of the vortical flow on the cells.

In this study, the cross‐slot chip demonstrates impressive bioproduction of EVs from spheroids composed of human mesenchymal stem cells while operating with a minimal number of producer cells. Moreover, thanks to the precise control of the hydrodynamic conditions, it provides a comprehensive exploration of the mechanistic underpinnings of EV release. Remarkably, it evidences that a brief yet intense stress on spheroids is sufficient to induce mass release of EVs.

## 2 Results and Discussion

### 2.1 High‐Throughput Spheroid Formation in Microwell Arrays for Bioproduction under Physiological Conditions with Adapted Vortical Flow

The objective of using the cross‐slot chip here was to mass‐produce EVs for regenerative medicine applications. The working hypothesis entailed translating the use of 2D culture on 200‐µm beads, as performed in bioreactors, to a 3D configuration consisting of 100–200 µm diameter spheroids composed solely of cells, ensuring heightened physiological relevance.^[^ ^51^ ^]^ Human mesenchymal stem cells (hMSCs) were selected with regards to their many advantages for the EVs they produce not only in tissue regeneration but also in cancer therapy, among other.^[^ ^65^, ^66^ ^]^ It is thus a now well‐established cell line model for bioproduction of therapeutic EVs. Note also that most study uses mouse derived MSCs, that were preferred here for human ones (hMSCs) for future clinical applications.

**Figure 1:**
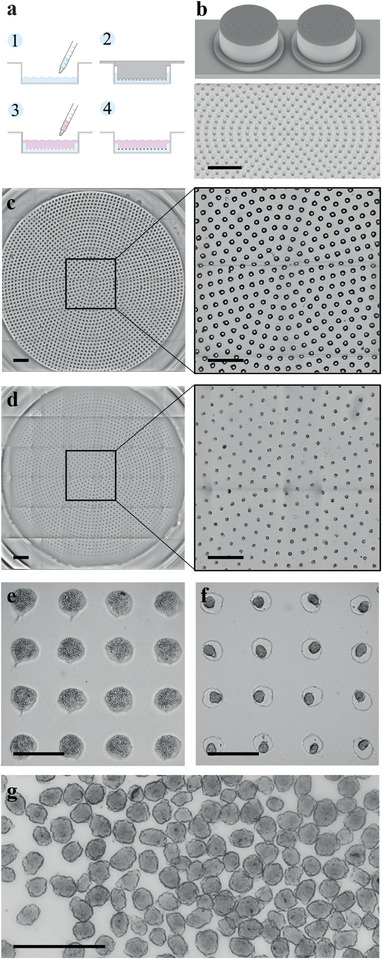
High‐throughput method for the formation of hMSC spheroids in a microwell array. a) Schematic representation illustrating the experimental protocol for the formation and maturation of hMSC spheroids. In step 1, a heated 2% agarose hydrogel is poured into a well of a 6‐well plate. Step 2 involves placing a mold exhibiting micropillars of 200 µm height and diameter onto the heated agarose, followed by 20 min of cooling at 4 °C. After agarose gelation, the mold is removed and a suspension of individualized hMSC is added to the well, followed by incubation at 37 °C for 24 h (Step 3). Step 4 depicts the resulting spheroid formed within the microwells after maturation. b) 3D view of the 3D‐printed microwell mold and zoomed‐in view of the micropillars array. Scale bar = 2 mm. c) Bright‐field microscopy image picturing the agarose microwells just after molding and before cell seeding. Scale bar = 2 mm. d) Bright‐field microscopy image picturing the agarose microwells with the cohesive hMSC spheroids after 24 h of maturation. Scale bar = 2 mm. e) Bright‐field microscopy image capturing hMSC cells within microwells following seeding (step 3). Scale bar = 500 µm. f) Bright‐field microscopy image capturing cohesive hMSC spheroids within microwells after 24 h of maturation (step 4). Scale bar = 500 µm. g) Bright field image of a suspension cohesive hMSC spheroids produced in the microwells. Scale bar = 2 mm.

The principle of spheroid formation is then straightforward: microwells of 200 µm in diameter and 200 µm in height are created using agarose molding, employing 3D‐printed stamps. **Figure** **1**a schematically illustrates the steps of the process: Cells previously detached from their culture flasks are deposited onto the micro‐structured agarose at a density of 300 cells per microwell and allowed to spontaneously sediment into the microwells. After 24 h of incubation, hMSCs matured into cohesive spheroids. To achieve high‐throughput spheroid production, circular molds were 3D‐printed, each exhibiting 2269 pillars and accommodated to fit into wells of a 6‐well plate (Figure 1b). Consequently, the entire plate generates 13614 spheroids. Figure 1c shows the agarose microwell array before cell seeding, while Figure 1d depicts the spheroids within the microwells after 24 h of maturation, with a zoomed‐in view on a specific region with hMSCs before (Figure 1e) and after (Figure 1f) spheroid maturation. Spheroids can then be easily retrieved by pipetting, and Figure 1g shows an image after harvesting. Their diameters were found to range from 50 µm to 175 µm, with an average diameter of 130 ± 15 µm.

### 2.2 Flow Dynamics and Spheroid Trajectory Analysis in the Cross‐Slot Chip

**Figure 2:**
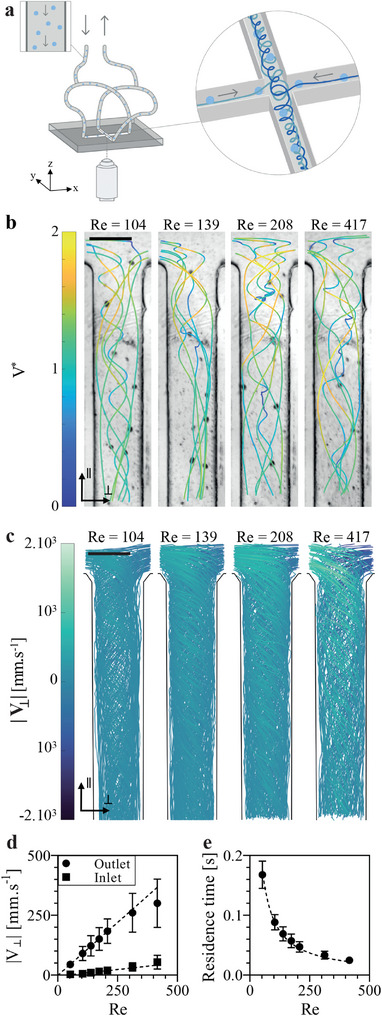
Controlled vortex formation within the cross‐slot chip. a) Schematic diagram of the experimental setup and the cross‐slot chip used for the study (b) Snapshots extracted from the tracking movies showing spheroid trajectories within the outlet channels. Trajectories are color‐coded based on the normalized velocity V^*^, calculated as the ratio between the magnitude of the projected velocity in the x‐y plane of the spheroid and the average velocity of the fluid. Scale bar = 1 mm. c) Spheroid trajectories within the outlet channels, color‐based on the magnitude of the orthogonal velocities of the spheroids projected in the x‐y plane. Scale bar = 1 mm. d) Average magnitude of the mean orthogonal velocities (in millimeter per second) along each trajectory plotted against Reynolds number. Circles and squares represent data from the outlet and inlet channels, respectively. Results are presented as mean ± SD (n = 80 – 295 depending on the condition). The dotted line indicates a linear fit for Re ≤ 208. e) Average residence time (in seconds) of the spheroids within the outlet channel plotted against Reynolds number. Results are presented as mean ± SD (n = 87 – 295 depending on the condition). The dotted line indicates a hyperbolic fit.

All spheroids from one plate were then typically resuspended in 5 mL of culture medium to achieve a density of 2400 spheroids per mL before being injected in the chip. This chip is composed of two opposing inlets and two opposing outlets, each with a length of 16 mm and square sections with a width of 1.2 mm, forming a cross‐junction (**Figure** **2**a). A suspension of hMSC spheroids was repeatedly circulated in this chip at increasing flowrates ranging from 7.5 to 60 mL min^−1^. These flow rates corresponded to Reynolds numbers (Re) ranging from 52 to 417 with

(1) $$Re=\frac{\rho wV_{0}}{\eta }$$

where ρ and η are respectively the density and the dynamic viscosity of the flowing fluid, w the width of the channel and V_0_ the average bulk velocity of the fluid.

Spheroids were recorded while transported both in the inlets and outlets for all studied Reynolds numbers, using a high‐speed camera and a microscope objective with a large depth of field of 25 mm, larger than the channel width. Spheroid trajectories could thus be followed everywhere in the channel. Video excerpts of the recordings are shown in Movie S1 (Supporting Information). 2D projections of spheroid trajectories were reconstructed from the recordings using image treatment. These trajectories result from the local interaction of the spheroids with the complex surrounding flow and display the flow conditions experienced during their transport. Due to the finite size of the spheroids their trajectories can however deviate from local flow velocities and streamlines.^[^ ^55^ ^]^

Figure 2b depicts spheroid trajectories for selected conditions in the outlet channels reflecting the different flow transitions occurring with increasing Reynolds numbers. Trajectories are colored with respect to norm of the local spheroid velocities projected in the x‐y plane and normalized by the average flow velocity. The complete results for all Reynolds numbers tested are shown in Figure S1 (Supporting Information). As it has been reported before,^[^ ^52^, ^53^, ^55^ ^]^ a symmetry breaking occurs around Re = 40, and a single steady‐state spiral vortex is observed in the center of the outlet channels. Tracking of hMSC spheroids at Re = 52 (Figure S1, Supporting Information) and Re = 104 (Figure 2b) indeed revealed spiral trajectories along the longitudinal axis, with radii strongly depending on the position of the spheroid in the channel. For Re ≥ 110 previous studies^[^ ^56^ ^]^ reported, in similar geometries, the emergence of two secondary counter rotating vortices and vortex breakdown leading to a zone of recirculation in the center of the channel. This perfectly fits with our observations for Re ≥ 139, where we witnessed increasing complexity of the trajectories, with the apparition of secondary vortices in the corners of the channel next to the central vortex (Figure 2b). The upstream transport of spheroids in the center of the outlet is visible in Figure S2 (Supporting Information). For Re>300 time‐dependent flow patterns have been predicted^[^ ^57^ ^]^ but are not directly visible from the spheroid trajectories shown here. However, the observed patterns get more and more irregular for large Reynolds numbers, in agreement with unsteady flow conditions.

We have no direct access to the local flow velocities in our experiments, but we can nevertheless gain important information from the spheroid trajectories. For stable flow in a straight channel, as is the case of the inlets, we expect straight trajectories and velocity components orthogonal to the bulk velocity to be zero. This is indeed the case up to very large Reynolds numbers where small deviations from straight trajectories are observed and a slight increase in average orthogonal velocity can be detected (Figure 2d). Measuring average longitudinal velocities along the tracks in the inlet channels revealed good agreement with the imposed average bulk velocity ensuring the quality of the tracking procedure (Figure S3, Supporting Information). To characterize the apparition of the vortex in the outlets we determined the norm of the orthogonal velocity component *V*_⊥_ in the x‐y plane as a function of Re. Non‐zero orthogonal velocity components can be seen for all experiments (Figure 2c; Figure S4, Supporting Information) in agreement with the existence of a vortex. The increase of the average *V*_⊥_ (Figure 2d) indicates, as expected, an increase in vorticity strength with increasing Re. Interestingly, while the average *V*_⊥_ appears to increase linearly for Re ≤ 209, it then deviates from this linearity from Re ≥ 315, coinciding with the apparition of unsteady flows at Re > 300.

Measuring the time spent by all tracked spheroids to cross the field of view allowed us to extrapolate a residence time of each spheroid in the outlet channel for all Re (Figure 2e). As expected, this average residence time per passage in the outlet is proportional to the inverse of the average velocity V_0_ and thus the inverse of Re, ranging from 168 milliseconds for Re = 52 to 25 milliseconds for Re = 417.

It should be noted that, as the trajectory of a spheroid is highly dependent on its entry point in the outlet channel, there is a significant heterogeneity in trajectories and residence times between the spheroids leading to a heterogeneity in the flow history they are submitted to during transport in the cross‐slot chip. The coefficient of variation of the orthogonal velocity (Figure 2d), which is ≈ 35%, and of the residence time (Figure 2e), which is ≈ 20%, illustrates this high heterogeneity. However, we can expect that recirculating multiple times the spheroid suspension in the chip leads to a reduction in this variability, resulting in a narrower distribution of residence time and flow history across the population.

Moreover, as only projections of spheroid trajectories were accessible in our set‐up, we were unable to access the full velocity field of the flowing fluid. Consequently, viscous stresses experienced by the spheroids along their trajectories, such as compressional, elongational or shear stresses, were impossible to derive. However, it is likely that the increase in vorticity and complexity of the trajectories with respect to Re led to an increase in overall stresses applied to the spheroids flowing into this chip.

### 2.3 Reynolds‐Dependent Mass Production of EVs from hMSCs Spheroids in the Cross‐Slot Chip

**Figure 3:**
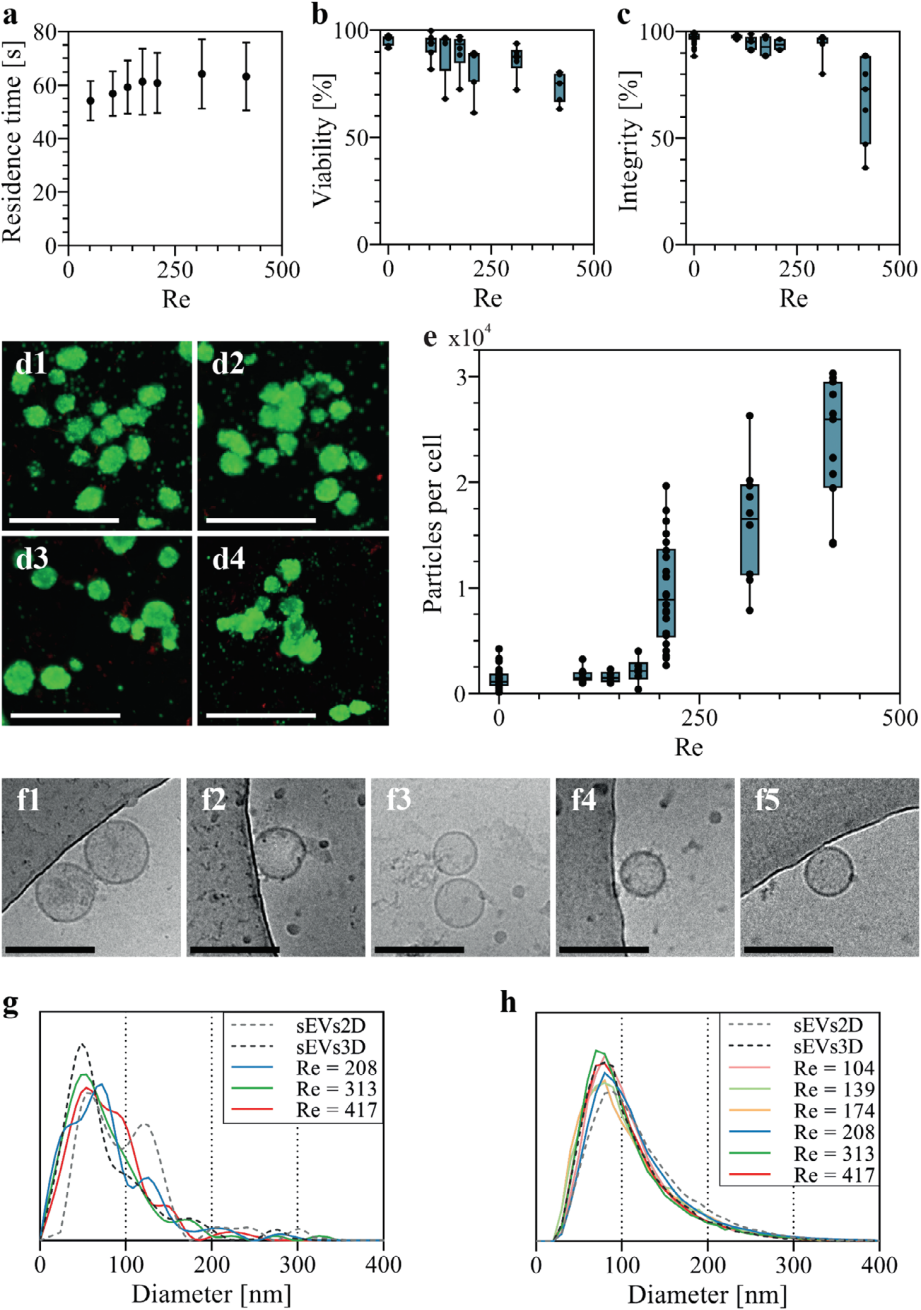
Hydrodynamic stimulation in a cross‐slot chip for mass production of extracellular vesicles from hMSCs spheroids while preserving cellular integrity. a) Total residence time (in seconds) of the spheroids within the outlet channel per hour of circulation in the cross‐slot chip, plotted against Reynolds number. Results are presented as mean ± SD (n = 87 – 205 depending on the condition). b) Measurement of hMSC spheroid integrity after 2 h of stimulation at various flow rates in the cross‐slot chip. Each data point represents an independent production experiment. c) Viability assessment by ToxiLight assay of hMSC spheroids after 2 h of stimulation at various flow rates in the cross‐slot chip. Each data point represents an independent production experiment. d) Live/Dead staining and imaging of **(d1)** unstimulated hMSC spheroids, **(d2)** hMSC spheroids stimulated in the cross‐slot chip at Re = 208, **(d3)** hMSC spheroids stimulated in the cross‐slot chip at Re = 313 and (d4) hMSC spheroids stimulated in the cross‐slot chip at Re = 417. Scale bars = 500 µm. e) Quantification of particles produced per cell after 2 h of stimulation in the cross‐slot chip, plotted against Reynolds number. Each data point represents an independent production experiment. f) Cryogenic transmission electron microscopy imaging of extracellular vesicles produced from (f1) serum‐deprived hMSC monolayers (sEVs2D), (f 2) serum‐deprived hMSC spheroids (sEVs3D) and hMSC spheroids stimulated in the cross‐slot chip at (f3) Re = 208, (f4) Re = 313 and (f5) Re = 417. g) Diameter distributions of EVs produced in 72 h by serum‐deprived hMSC monolayers (sEVs2D) and hMSC spheroids (sEVs3D), and by spheroids stimulated for 2 h in the cross‐slot chip at Re = 208, 313 and 417, assessed via cryoTEM imaging (n = 49 – 250 depending on the condition). Curves were smoothed using a cubic spline interpolation. h) Diameter distributions of EVs produced in 72 h by serum‐deprived hMSC monolayers (sEVs2D) and spheroids (sEVs3D), and by spheroids stimulated for 2 h in the cross‐slot chip (Re = 104 – Re = 417), measured by NTA (n = 14103 – 157952 depending on the condition). Curves were smoothed using a cubic spline interpolation.

For bioproduction, hMSC spheroids suspended at a density of 2400 per mL were subjected to repeated circulations within the chip for a duration of 2 h, under controlled 37 °C condition. Flow rates were tested at 15, 20, 25, 30, 45, and 60 mL min^−1^, corresponding to Reynolds numbers spanning from 104 to 417, as described above. Within this range, the entire volume of spheroid suspension circulated between 1286 and 5143 times through the chip. However, experimental measurements of residence time in the outlet channel revealed a proportionality to the inverse of the Reynolds number (Figure 2e). Consequently, despite the variations in flow rates, the total time spent by the spheroids within the high‐stress zone inside the vortical flow remained relatively constant across the entire range of Re, averaging ≈60 s h^−1^ of circulation (**Figure** **3**a).

The biological impact of the 2‐h production in the cross‐slot chip was evaluated at the spheroids level. Figure 3b displays the percentage of intact spheroids following repeated circulations and demonstrates the absence of damage for Reynolds numbers ≤ 313. A decrease in integrity was solely observed at Re = 417, with cohesiveness ranging from 88% to 36%. Cellular viability was further assessed using Toxilight assay (Figure 3c). Viability remained unaltered compared to unstimulated spheroids for Re ≤ 174, with over 90% of viable cells. However, viability started to decline at Re = 208, culminating in ≈25% mortality at Re = 417. These findings were substantiated by Live/Dead staining of the spheroids (Figure 3d), revealing predominantly viable cells under all tested conditions, albeit with a growing proportion of dead cells observed for Re ≥ 208. Those dead cells were rarely localized on intact spheroids, but rather on spheroid debris, aligning with integrity measurements, as more individualized cells were observed at Re = 417.

After the spheroids were recirculated in the chip for 2 h, the circulating medium was collected and centrifuged at 2000 g for 10 min to eliminate not only the spheroids but also all individual cells, debris, or apoptotic bodies. The supernatant, also known as conditioned medium (CM), therefore contained the EVs released by the spheroids. Particles concentration in CM was then quantified by Nanoparticle Tracking Analysis (NTA) (Figure 3e). Remarkably, it clearly elucidates the presence of a threshold in production within a range of Reynolds numbers between 174 to 208. Below this threshold, particle release was similar to that of unstimulated spheroids kept for 2 h in a 37 °C environment (Re = 0), whereas above it, significant particle release was achieved, with enhancements of tenfold, 15‐fold and 20‐fold compared to unstimulated spheroids, for Re of 208, 313, 416, respectively. High flow rates, which generate intensified viscous stresses, undeniably augment particle release. Remarkably, this threshold does not correlate with any observed or described alterations in flow characteristics as described above; instead, it aligns with a linear increase in orthogonal velocity (Figure 2d). Additionally, no particle release was observed in control experiments conducted using a straight channel chip with dimensions and length similar to those of the cross‐slot chip, even at a Reynolds number of 208 (Figure S5, Supporting Information). This demonstrates that particle production is attributable to the high stress zone associated with the vortex flow within the cross‐slot outlet channel, rather than the shear flows generated throughout the recirculation system or the straight channel parts of the chip. Saturation in particle production was not attained within the investigated range of Reynolds numbers, as limited by the feasible conditions achievable experimentally. At the highest Reynolds number exceeding 400, productions yielded an average of 30 000 particles per cell, emphasizing the efficacy of the cross‐slot chip in enabling substantial particle production. However, what is most remarkable is that this level of particle release was achieved within just 2 min of stress application in the spiral vortex flow (equivalent to 60 s per hour of recirculation, as illustrated in Figure 3a). At this stage, it remains unclear whether particles are exclusively produced during this high‐shear stimulation duration, or if it triggers particle release from spheroids that continue to produce within the chip during recirculation. If release solely occurs only during stimulation, it would be equivalent to 15 000 EVs released per minute. For comparison, current high‐yield techniques of EV production release EVs continuously over hours and yield ≈ 5000; 10 000; 20 000; and 30 000 EVs released per hour for methods such as vertical wheel; hyperflasks; extrusion; and quantum hollow‐fiber bioreactor as well as spinner flasks operating within a turbulent regime, respectively. The yield in the cross‐slot chip could therefore impressively be 30‐fold higher than the most efficient other techniques. However, this comparison should be approached with caution because, just as we cannot ascertain whether production here could be vortex‐triggered and then continued, similarly, it cannot be determined if production is continuous or intermittent in other techniques.

Cryogenic transmission electron microscopy (cryoTEM) confirmed the vesicular nature and structural integrity of the particles produced under 72‐h starvation from monolayers (sEVs2D) or spheroids (sEVs3D), as well as those produced hydrodynamically in the cross‐slot chip from spheroids (Figure 3f; Figure S6, Supporting Information). The diameters of all observed EVs are presented in Figure 3g, showing a mean diameter of 80 nm and a polydispersity index of 0.6 when produced from spheroids, regardless of whether they were generated under 72‐h starvation conditions (sEVs3D) or within the cross‐slot chip under different stimulation conditions (Re = 208, 313, 417). EVs released from 2D‐monolayers (sEVs2D) under starvation conditions were similar, though with a slightly larger mean diameter of 100 nm with a polydispersity index of 0.7. Size distribution analysis using NTA (Figure 3h) indicated that EVs produced from spheroids, whether through 72‐h serum deprivation (sEVs3D) or hydrodynamic stimulation (Re = 104 – 417), had similar diameter distributions with mean diameters ≈100 nm. EVs released from serum‐deprived monolayers (sEVs2D) exhibited slightly larger diameters, averaging ≈120 nm. Overall, both cryoTEM and NTA measurements indicate that EVs generated from hMSCs spheroids exhibit nearly identical size distributions regardless they were produced under serum starvation or through in‐chip stimulation. Those produced from monolayers are similar, with a slightly larger average diameter.

### 2.4 Cross‐Slot Chip Not Only Facilitates Efficient EV Production but Also Imparts Distinct Biological Signature

**Figure 4:**
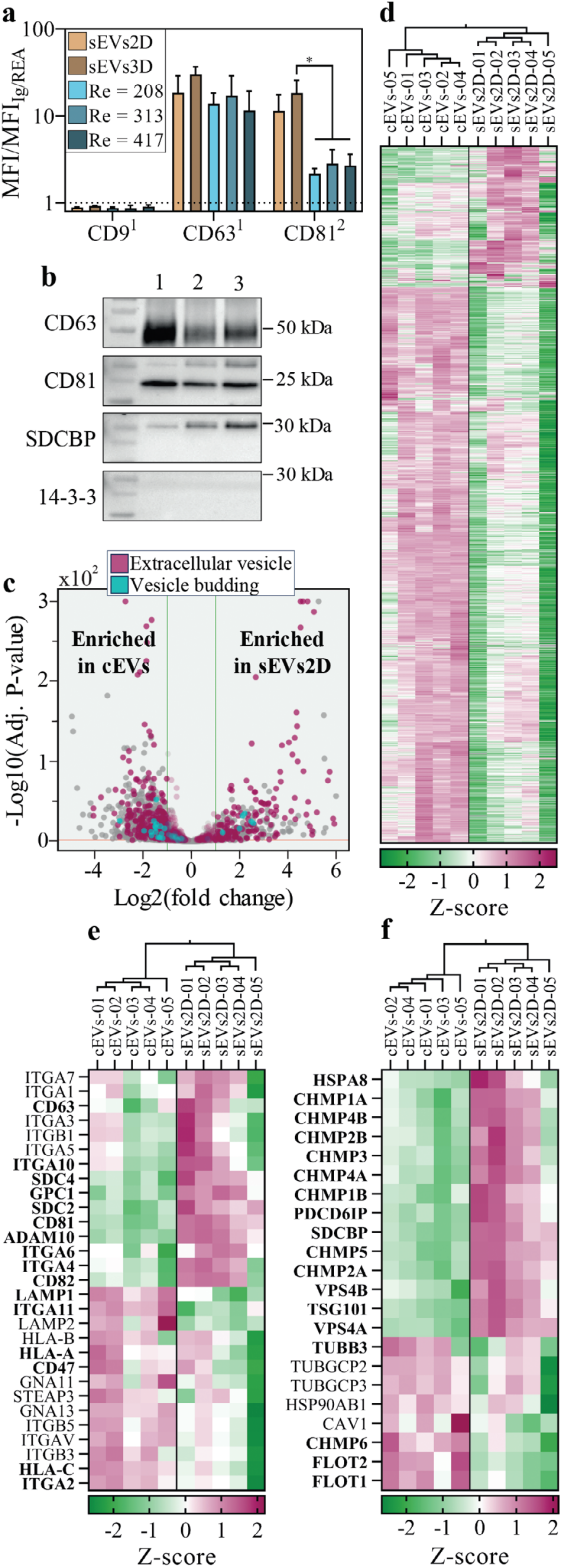
Unique biological signature of extracellular vesicles produced in a cross‐slot chip. a) Bead‐based fluorescence flow cytometric analysis of EV‐specific protein markers in extracellular vesicles produced from unstimulated hMSC (sEVs2D), unstimulated hMSC spheroids (sEVs3D) and hMSC spheroids stimulated in the cross‐slot chip at Re = 208, 313 and 417. Results are presented as ratios of mean fluorescence intensity with respect to mIgG1 (1) or REA (2) controls. Results are shown as mean ± SD (n = 3). ^*^ = *p* < 0.05 (unpaired two‐tailed Student's *t*‐test). b) Western blot analysis of specific EV membrane (CD63, CD81) and cytosolic (SDCBP or Syntenin‐1) markers, along with a co‐isolated non‐EV marker (14‐3‐3) for EVs produced from hMSC spheroids stimulated in the cross‐slot chip at Re = 208 (1) and for EVs produced through serum starvation from hMSC in 2D configuration (2) and hMSC spheroids (3). c) Proteome comparison of EVs produced through serum starvation from hMSC in 2D configuration (sEVs2D) and EVs produced from hMSC spheroids stimulated in the cross‐slot chip at Re = 313 (cEVs) described in a volcano plot. Proteins belonging to the “extracellular vesicle” GO term (GO:1903561) and to the “vesicle budding from membrane” GO term (GO:0006900) are shown in red and blue, respectively, from the total of 4459 proteins quantified (Table S1, Supporting Information). Only proteins quantified in both conditions are shown (proteins quantified in only one of the two conditions are listed in Table S2, Supporting Information). d) Heatmap with hierarchical clustering of EVs produced through serum starvation from hMSC in 2D configuration (sEVs2D) and EVs produced from hMSC spheroids stimulated in the cross‐slot chip at Re = 313 (cEVs), showing Z‐scores of all the proteins found in both conditions. e) Heatmap with hierarchical clustering of EVs produced through serum starvation from hMSC in 2D configuration (sEVs2D) and EVs produced from hMSC spheroids stimulated in the cross‐slot chip at Re = 313 (cEVs), showing Z‐scores of transmembrane EV markers as described in the MISEV.^[^ ^68^ ^]^ Proteins with significantly different levels between the two conditions (Fold change > 2 and adjusted p‐value < 0,05) are highlighted in bold. f) Heatmap with hierarchical clustering of EVs produced through serum starvation from hMSC in 2D configuration (sEVs2D) and EVs produced from hMSC spheroids stimulated in the cross‐slot chip at Re = 313 (cEVs), showing Z‐scores of cytosolic EV markers as described in the MISEV.^[^ ^68^ ^]^ Proteins with significantly different levels between the two conditions (Fold change > 2 and adjusted p‐value < 0,05) are highlighted in bold.

EV‐specific markers were quantified using bead‐based fluorescence flow cytometry (MACSPlex Exosome Kit) and compared with a standard EV production method involving serum starvation (**Figure** **4**a). Briefly, EVs were released through 72 h of serum starvation by hMSC in a 2D monolayer (sEVs2D) or 3D spheroids (sEVs3D) configuration, or by hMSC spheroids stimulated for 2 h within the cross‐slot chip at different Reynolds numbers (Re = 208, 313 and 417). Across all conditions, the EV‐specific marker CD63 was consistently expressed. Notably, CD81 was enriched in EVs from serum‐starved conditions, with statistically significant differences observed only between the sEVs3D condition and the cross‐slot chip conditions. All EVs also tested negative for CD9, which is known to exhibit variable expression in human mesenchymal stem cells.^[^ ^67^ ^]^

Western blot analysis of EVs produced from serum‐deprived hMSCs in 2D configuration, serum‐deprived hMSC spheroids and hMSC spheroids stimulated in the cross‐slot chip at Re = 313 (Figure 4b) also confirmed the presence of specific EV markers, including the transmembrane proteins CD63 and CD81, as well as the cytosolic protein SDCBP (or syntenin‐1) under all tested conditions. Importantly, all EV samples lacked the co‐isolated non‐EV marker 14‐3‐3, thus validating the nature and the purity of the isolated EVs.

To investigate the difference in protein cargo between EVs produced through serum starvation (sEVs2D) and EVs produced in the cross‐slot chip (cEVs, Re = 313), a comprehensive proteomic analysis was conducted using quantitative label‐free mass spectrometry on five independent biological replicates (independent initial culture of hMSC prior to production). A total of 6643 proteins were successfully detected in at least one replicate and 4459 proteins were quantified from at least 2 distinct peptides across all five replicates of one of the two conditions (Table S1, Supporting Information), including 3025 proteins detected in both conditions with an adjusted p‐value below 0.05 of the fold change. The volcano plot (Figure 4c) and the Z‐score heatmap (Figure 4d) of these 3025 proteins, as well as the principal component analysis (PCA) (Figure S7, Supporting Information), indicate that EVs from these two different conditions have distinct proteomic profiles. PCA also evidenced a higher homogeneity between replicates of the cEVs condition, suggesting a better batch‐to‐batch consistency for the cross‐slot chip production method. Notably, 326 proteins were significantly enriched (Log2 fold change > 2 and adjusted p‐value < 0.05) in the sEVs2D, while 1248 proteins were enriched in the cEVs. Interestingly, a total of 796 proteins were associated with the Gene Ontology term “extracellular vesicle” (GO:1903561), including 261 proteins significantly enriched in the cEVs condition and 148 proteins significantly enriched in the sEVs2D condition (Figure 4c). Identically, a total of 44 proteins were associated with the Gene Ontology term “vesicle budding from membrane” (GO:0006900), including 16 proteins significantly enriched in the cEVs condition, and 6 proteins significantly enriched in the sEVs2D condition (Figure 4c). Further GO term analysis in the subset of proteins enriched in the EVs produced in the cross‐slot chip exhibited an enrichment factor of 1.97 for the GO term “extracellular vesicle” (adjusted p‐value = 4.28e‐26) and of 3.53 for the GO term “vesicle budding from membrane” (adjusted p‐value = 2.59e‐4). Additionally, 25 and 46 proteins, were quantified exclusively in the sEVs2D and in the cEVs conditions, respectively (Table S2, Supporting Information).

Specific attention was given to EV markers as outlined in the Minimal information for studies of extracellular vesicles (MISEV2023) guidelines.^[^ ^68^ ^]^ Of the 60 proposed transmembrane markers and 47 cytosolic markers, 29 and 22, respectively, were found in significant quantities in the proteome analysis. Heatmaps of Z‐score for both transmembrane markers (Figure 4e) and cytosolic markers (Figure 4f) show notable differences in proteomic profiles between the two conditions. Notably, all markers tested by fluorescence flow cytometry (Figure 4a) and western blot (Figure 4b) were found in both conditions, with CD63, CD81 and SDCBP significantly enriched in the sEVs2D condition. Strikingly, Z‐scores of detected cytosolic markers suggest different biogenesis pathways for the two different stimulations. EVs produced from 2D cultured hMSC through serum starvation were indeed found to be significantly enriched in exosome‐related proteins (PDCD6IP, TSG101),^[^ ^69^ ^]^ and EVs produced from hMSC spheroids hydrodynamically stimulated in the chip significantly enriched in microvesicle‐related proteins (tubulin,^[^ ^70^ ^]^ flotillin^[^ ^69^ ^]^).

### 2.5 EVs Generated Within the Cross‐Slot Chip Exhibit Regenerative Potential

**Figure 5:**
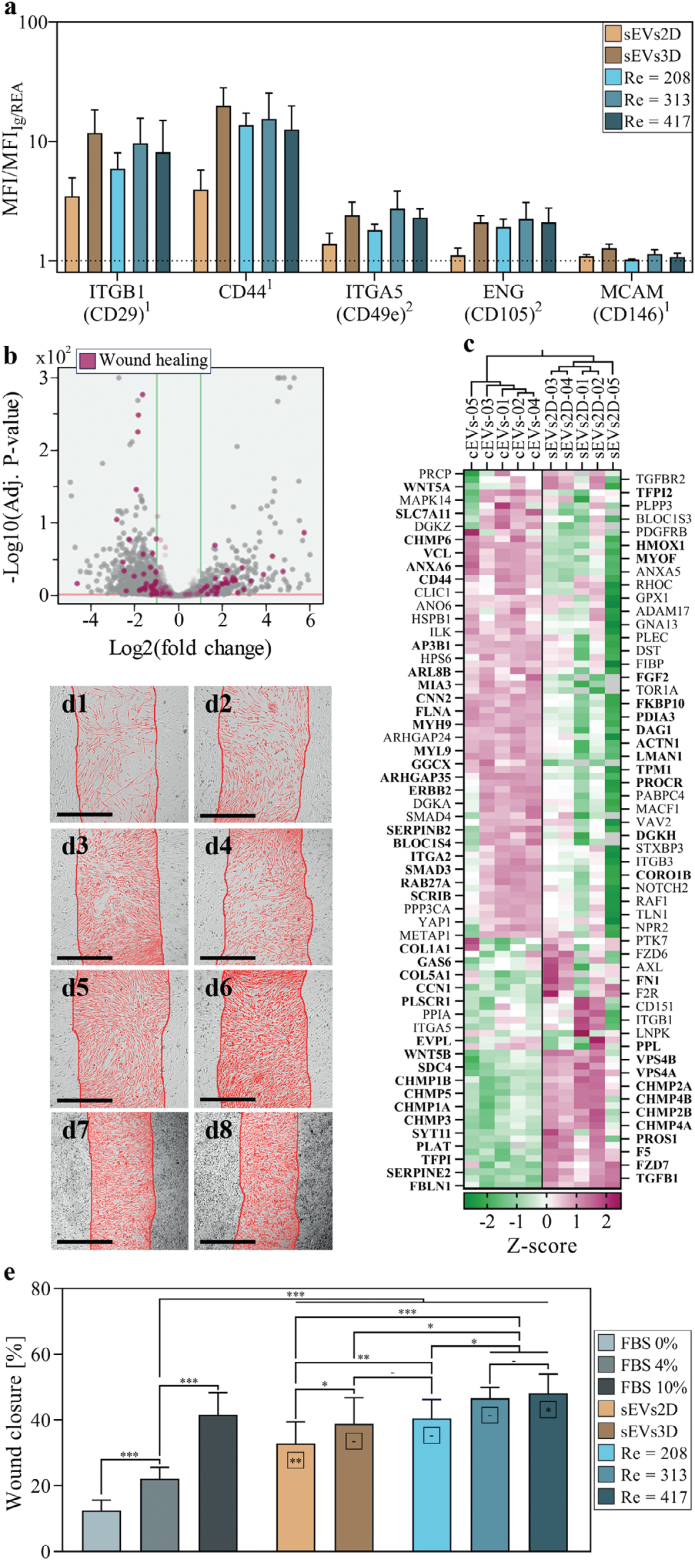
Enhanced wound healing properties. a) Bead‐based fluorescence flow cytometric analysis of mesenchymal‐specific protein markers in extracellular vesicles produced from unstimulated hMSC (sEVs 2D), unstimulated hMSC spheroids (sEVs 3D) and hMSC spheroids stimulated in the cross‐slot chip at Re = 208, 313 and 417. Results are presented as ratios of mean fluorescence intensity with respect to mIgG1 (1) or REA (2) controls. Results are shown as mean ± SD. (n = 3) (b) Volcano plot illustrating the differential protein composition between EVs produced through serum starvation from hMSC in 2D configuration (sEVs2D) and EVs produced from hMSC spheroids stimulated in the cross‐slot chip at Re = 313 (cEVs) described in a volcano plot. Proteins belonging to the “wound healing” GO term (GO: 0042060) are shown in red, from the total of 4459 proteins quantified (Table S1, Supporting Information). Only proteins quantified in both conditions are shown (proteins quantified in only one of the two conditions are listed in Table S2, Supporting Information). c) Heatmap with hierarchical clustering of EVs produced through serum starvation from hMSC in 2D configuration (sEVs2D) and EVs produced from hMSC spheroids stimulated in the cross‐slot chip at Re = 313 (cEVs), showing Z‐scores of the proteins belonging to the “wound healing” GO term (GO:0042060). Unassigned proteins are shown in grey. Proteins with significantly different levels between the two conditions (Fold change > 2 and adjusted p‐value < 0,05) are highlighted in bold. d) Bright‐field images showing the wound scratches after 72 h of incubation in (d1) serum‐deprived culture medium, or culture medium supplemented with (d2) 4% FBS, (d3) 10% FBS, (d4) EVs produced through serum starvation from hMSC in 2D configuration, (d5) EVs produced through serum starvation from hMSC spheroids and (d6) EVs produced from hMSC spheroids stimulated in the cross‐slot chip at Re = 313. e) Wound closure after 72 h of incubation, calculated as the area covered by human skin fibroblasts in the wound, for cells incubated in serum‐deprived culture medium (FBS 0%) or culture medium supplemented with 4% or 10% FBS, EVs produced through serum starvation from hMSC in 2D configuration (sEVs 2D) or hMSC spheroids (sEVs 3D) and EVs produced from hMSC spheroids stimulated in the cross‐slot chip at Re = 208, Re = 313 or Re = 417. Results are shown as mean ± SD (n = 6 – 15 depending on the condition). Statistical significances showed in boxes are related to the FBS 10% condition. – = non‐significant, ^*^ = *p* < 0.05, ^**^ = *p* < 0.01, ^***^ = *p* < 0.001 (unpaired two‐tailed Student's *t*‐test).

In all condition, EVs expressed mesenchymal‐specific markers ITGB1 (CD29), CD44, ITGA5 (CD49e), and ENG (CD105), but not MCAM (CD146), which is unequally expressed among subpopulations of hMSCs (**Figure** **5**a).^[^ ^71^ ^]^ Additionally, the levels of these markers were higher in EVs generated from spheroids, both through serum starvation and hydrodynamic stimulation, compared to those originating from 2D monolayers. This observation was confirmed by quantitative proteome analysis, further validating the enhanced biological relevance of employing a 3D model. All EVs tested negative for hematopoietic‐associated and for endothelial‐associated markers (Figure S8, Supporting Information).

Proteome analysis of EVs produced from 2D monolayers through serum starvation (sEVs2D) and EVs produced from spheroids in the cross‐slot chip (cEVs) revealed distinct profiles for the 109 proteins associated with the Gene Ontology term “wound healing” (GO:0042060) which were detected at significant levels. The volcano plot (Figure 5b) and the Z‐score heatmap (Figure 5c) exhibited 31 and 36 proteins, respectively, significantly enriched (Log2 fold change > 2 and *p* < 0.05) in the sEVs2D condition and in the cEVs condition. Further GO term analysis in the subset of proteins enriched in the EVs produced in the cross‐slot chip exhibited an enrichment factor of 2.25 for the GO term “wound healing” (adjusted *p*‐value = 4.02e‐16).

Finally, the functional capabilities of EVs derived from hMSC spheroids, were specifically assessed through wound healing and neo‐angiogenesis assays.

The wound healing assay was conducted using EVs produced under starvation conditions from hMSCs in monolayer (sEVs2D) and spheroid (sEVs3D) configurations, as well as EVs released from hMSC spheroids stimulated within the cross‐slot chip at different Reynolds numbers (Re = 208, 313 and 417). The assay was performed using human skin fibroblasts (HSF) seeded into 96‐well plates. A physical gap was created within the monolayer, and the migration of fibroblasts into the gap was monitored following the administration of medium alone, or medium supplemented with EVs preparations or serum (4% or 10% FBS). Gap closure was tracked for a period of 72 h (Movie S2, Supporting Information) and quantified by measuring the total area covered by cells. Representative images illustrating wound healing at the 48‐h time point are depicted in Figure 5d, while Figure 5e presents the percentage of wound closure. As expected, increasing FBS concentration enhanced wound closure. EVs released from spheroids, both in static conditions under serum starvation and through in‐chip hydrodynamic stimulation, exhibited superior healing capacities compared to EVs produced from 2D monolayers under starvation (control condition), highlighting the relevance of 3D models. This includes EVs produced in‐chip at Re = 313, whose wound healing‐related proteomic profile showed significant differences compared to the control condition, as shown in Figure 5b,c. While EVs produced through hydrodynamic stimulation at low Reynolds (Re = 208) demonstrated similar healing abilities to those from serum‐starved spheroids (sEVs3D), increasing the Reynolds number in the chip (Re = 313 and Re = 417) enhanced their wound healing capabilities (p = 0.033 and p = 0.018, respectively). Notably, while EVs produced from spheroids in static conditions upon serum starvation and from hydrodynamically stimulated spheroids at Re = 208 and Re = 313 achieved wound closure comparable to the FBS 10% positive control, only the EVs produced at Re = 417 exceeded the wound closure observed with the FBS 10% condition. Additionally, measurements taken at earlier time points of 24 and 48 h (Figure S9, Supporting Information) showed that all EV‐treated conditions outperformed the FBS condition, particularly at 24 h. Overall, wound healing was more efficient with EVs produced from hMSC spheroids, particularly those stimulated in the cross‐slot chip at intermediate (Re = 313) or high (Re = 417) Reynolds, underscoring the therapeutic potential of the cross‐slot chip for EV production.

Neo‐angiogenesis assay was performed using human umbilical vein endothelial cells (HUVEC) spheroids embedded in collagen and incubated for 24 h with either EVs produced from serum‐deprived hMSC spheroids (sEVs3D), or with EVs produced from hMSC stimulated in the chip at Re = 313 (cEVs), as well as with culture medium supplemented with vascular endothelial growth factor (VEGF‐α) at different concentrations. The quantitative analysis of sprout formation by the spheroids (Figure S10, Supporting Information) demonstrated a significant increase in sprout quantity and length for all EV and VEGF conditions compared to the control.

### 2.6 Vortex Control: A Leap Forward in the Research Field of EV Bioproduction

This work distinguishes itself from other bioproduction methods by enabling precise control of EV release via stimulation in a flow environment. The hydrodynamic‐based innovation not only results in a significant increase in the yield of biologically active EVs from hMSC but also introduces the capability to precisely control the viscous forces applied. This enabled the discovery of a critical threshold necessary to trigger EV production.

This flow‐level control, unparalleled in its precision, markedly outperforms other methods. The ability to finely tune the production process addresses a long‐standing need^[^ ^72^ ^]^ within the scientific community for a more efficient and robust approach to EV production. Shifting to a pharmaceutical manufacturing perspective, the hydrodynamic flow features can be considered as critical process parameters (CPP), which means a key variable affecting the production process. CPP must be monitored or controlled because they have an impact on a critical quality attribute (CQA) of the product. The regulatory guideline ICH Q8 (R2)^[^ ^73^ ^]^ highlights the importance of the design space, which describes the relationship between the process inputs (i.e., process parameters) and the CQA. In this regard, we anticipate that the approach proposed herein will expedite future pharmaceutical manufacturing in terms of setting accurate CPP and design space. Indeed, the journey toward the regulatory approval and the commercialization of EV‐based therapies is marked by the unmet need for more efficient and controllable EV bioproduction processes. The path to the market is shaped by market dynamics, where on one hand, drivers such as extensive academic research on EVs propel the field forward. On the other hand, technological challenges associated with the implementation of efficient, robust, and controllable EV bioprocesses pose significant market restraints, as evidenced by market analyses.^[^ ^74^, ^75^ ^]^ In this context, achieving precise control and monitoring over viscous stresses to trigger substantial EV release via brief, intense stimulation represents a pivotal strategy. Such an approach may bridge academic research with regulatory requirements and market demand to successfully transition innovative therapies from the lab to the market.

## 3 Conclusion

Hydrodynamic stimulation of hMSC spheroids within the cross‐slot chip demonstrated remarkable effectiveness in inducing substantial and robust bioproduction of biologically active EVs. Major advancements in the field of EV bioproduction include 1) exploiting and controlling single vortex formation in the cross‐slot geometry to trigger EV release, 2) adapting the system to support hMSCs in physiological 3D spheroid configurations, releasing EVs with mesenchymal signature when passing through the vortex, 3) operating with working volumes of a few milliliters, aligning with cell density requirements for precision medicine applications, and 4) precisely controlling flow fields through the Reynolds (Re) number and mapping experimentally the trajectories of individual spheroids within the vortex, opening the possibility to further evaluate the stress experienced by individual spheroids using for example numerical simulations. This last achievement enabled the determination of a Re‐dependent threshold for initiating EV release and revealed the temporal dynamics of EV secretion, demonstrating that brief yet intense stress‐induced stimulation triggers substantial and sustained EV bioproduction.

## 4 Experimental Section

### Cell Culture

hTERT‐immortalized human Mesenchymal Stem Cells (hMSC; abm, T0523), Human Umbilical Vein Endothelial Cells (HUVEC; ATCC, CRL1730) and Human Skin Fibroblasts (HSF; abm, T0326) were cultured at 37 °C in a 5% CO2 atmosphere using T150 (TPP, 90150), T75 (TPP, 90075) and PriCoat T25 (abm, G299) culture flasks, respectively. hMSCs were cultured in Prigrow II medium (abm, TM002) supplemented with 10% Fetal Bovine Serum (FBS) (Gibco, 12662029), 1% penicillin, and streptomycin (Gibco, 15140‐122) and 10^−6^ mol L^−1^ hydrocortisone (Sigma, H0135). HUVEC were cultured in DMEM (Gibco, 21885) supplemented with 10% heat‐inactivated FBS (Dutscher, S1900‐500C) and 1% penicillin and streptomycin (Gibco, 15140‐122). HSF were cultured in Prigrow III medium (abm, TM003) supplemented with 10% FBS (Gibco, A5209402) and % penicillin and streptomycin (Gibco, 15140‐122).

### Microwells Fabrication

Microwells circular molds were 3D‐printed, each exhibiting 2269 pillars of height and diameter of 200 µm and accommodated to fit into wells of a 6‐well plate. Molds were 3D‐printed using a DigitalWax 028J Plus 3D and the DigitalWax DS3000 resin. A solution of heated 2% agarose (Sigma, A0576) hydrogel in PBS was poured into the wells of a 6‐well‐plate, the molds were carefully positioned in contact with the agarose, and the entire assembly was placed at 4 °C for 20 min to facilitate the gelation of the agarose hydrogel. Following gelation, molds were carefully removed, and the plates were sterilized for 1 h under UV light and stored at 4 °C in 220‐nm filtered PBS for subsequent use.

### hMSC and HUVEC Spheroid Formation

hMSC from passage 10–20 or HUVEC from passage inferior to 30 were detached from their culture flasks, centrifuged at 300 g for 5 min and resuspended at a concentration of 700000 cells mL^−1^ in DMEM (Gibco, 61965‐026 for hMSCs and Gibco, 21885 for HUVEC) supplemented with 10% serum (Dutscher, S1900‐500C) and 1% penicillin/streptomycin (Gibco, 15140‐122). 6‐well plates containing the agarose microwells were then rinsed with PBS, and 1 mL of the cell suspension was deposited onto the microwells. Plates were left at rest 30 min at 37 °C to allow cell sedimentation, then centrifuged at 200 g for 2 min before being incubated at 37 °C for 24 h to generate cohesive hMSC spheroids, and 72 h to generate cohesive HUVEC spheroids.

### hMSC Spheroid Imaging and Harvesting

Following maturation, spheroids were imaged in their microwells using a plate reader in bright‐field mode (Perkin‐Elmer, Ensight Multimode Plate Reader). The average diameter of the spheroids was then assessed with FiJi software. hMSC spheroids were subsequently recovered from the microwells by pipetting. They underwent three washing steps through centrifugation (200 g, 3 min) and resuspension in 220‐nm filtered DMEM without phenol red (Gibco, 31053‐028). Spheroids were then resuspended in 220‐nm‐filtered DMEM without phenol red at a concentration of 2400 spheroids mL^−1^.

### Cross‐Slot Chip and Straight‐Channel Chip Fabrication

Cross‐slot chip and straight‐channel chip were designed as channels of 32 mm in length, 1.2 mm in width, and 1.2 mm in height. Cross‐slot chip featured two perpendicular channels intersecting at their midpoints, while straight‐channel chip featured a single channel. Both chips were made with 3D‐printed molds using the DigitalWax 028J Plus 3D‐printer and the DigitalWax DS3000 resin. Unpolymerized polydimethylsiloxane (PDMS), prepared by combining SYLGARD 184 Silicone Elastomer Base and SYLGARD 184 Silicone Elastomer Curing Agent in a mass ratio of 5:1, was poured into the molds. To avoid the formation of air bubbles, the PDMS‐filled molds were degassed and then incubated at 70 °C for 24 h to allow facilitate PDMS polymerization. After polymerization, the PDMS layers were carefully unmolded, and 2 mm inlets and outlets were punched at the ends of the channels. PDMS layers and 50 × 50 mm glass slides were cleaned with isopropanol and surface‐activated through plasma treatment using the Diener PICO plasma cleaner. Chips were then immediately assembled by placing the PDMS layer on the glass slide. Opposite outlets of cross‐slot chip were connected to each other using silicone tubing (length = 100 mm, inner diameter = 0.79 mm, outer diameter = 2.36 mm) and polypropylene T‐connectors. T‐connectors third outlets were then connected to another silicone tubing (length = 150 mm) at the end of which needles (diameter = 1.2 mm) were affixed to allow the connection to a syringe. Both outlets of straight‐channel chip were connected to a silicone tubing (length = 250 mm) at the end of which needles were affixed. Tightness of all connections was ensured with silicone coating (3140 RTV Coating, Dowsil). Hydraulic resistance of the system was finally meticulously tuned to ensure symmetrical flows in the cross‐slot chip, using a 3D‐printed ring designed to exert pressure on the tubing.

### hMSC Spheroid Tracking in the Cross‐Slot Chip

A total of 4 mL of the previously prepared spheroid suspension was distributed into UV‐sterilized 25 mL air‐tight syringes (Hamilton, 1025TLL). These syringes were then connected to the needles of the chips and affixed to a syringe pump (Cetoni GmbH, Base module 120, and Low‐Pressure Nemesys modules), controlled by the QmixElements software. The cross‐slot chip was placed under an inverted microscope (Leica, DM IRB) connected to a high‐speed camera (Phantom VEO‐E 310L), and the spheroid suspension was subjected to repeated circulations within the cross‐slot chip, with flow rates of 7.5, 15, 20, 25, 30, 45 and 60 mL min^−1^, corresponding to Reynolds numbers of 52, 104, 139, 174, 208, 313 and 417, respectively. Spheroid trajectories were recorded with an objective of magnification 2.5X and depth of field of 25 mm, with recording speed ranging from 6400 to 9600 images per second, depending on the in‐chip flowrate. The subsequent analysis of spheroid trajectories was performed using the TrackMate plugin in FiJi software. Particle detection was executed using the LoG detector method, with a quality threshold set at 10 and estimated object size of 0.025 mm. Particle tracking was then performed using the simple LAP Tracker method, with a linking max distance ranging from 0.05 to 0.15 mm depending on the in‐chip flowrate, and a gap‐closing max distance set as two times the linking max distance. Any incomplete or incorrect tracks were manually addressed by filling or correcting them using the Trackscheme tool. Velocities and residence times were derived from the tracked coordinates using Matlab. The density and the dynamic viscosity of the flowing fluid were considered equal to 103 g mL^−1^ and 10–3 Pa.s, respectively.

### EV Production Within the Cross‐Slot Chip

A total of 4 mL of the previously prepared hMSC spheroid suspension was distributed into UV‐sterilized 25 mL air‐tight syringes. These syringes were then connected to the needles of the chips and affixed to a syringe pump, controlled by the QmixElements software. Successive cycles of alternating back‐and‐forth movements were applied for 2 h to the syringes, with flow rates of 15, 20, 25, 30, 45, and 60 mL min^−1^, corresponding to Reynolds numbers of 104, 139, 174, 208, 313 and 417, respectively. Unstimulated spheroids were placed in a 2% agarose‐coated T25 flask as control. Following stimulation, spheroid suspensions were recovered and centrifuged at 200 g for 3 min to separate spheroid pellets from the EV‐conditioned media (CM). EV CM were then centrifuged at 2000 g and 4 °C for 10 min before being stored at 4 °C for further use. Spheroid pellets were resuspended in 400 µL of 220‐nm‐filtered DMEM without phenol red and stored at room temperature before further use.

### EV Production from 2D hMSC through Serum Starvation

hMSC were cultured in T150 flasks (TPP, 90150) as described above until confluency, and the culture medium was replaced by 20 mL of fresh serum‐deprived culture medium. The flasks were then incubated at 37 °C, 5% CO2 for 72 h. Following the EV production, EV‐conditioned media were recovered and centrifuged at 2000 g and 4 °C for 10 min before being stored at 4 °c for further use.

### EV Production from hMSC Spheroids through Serum Starvation

T150 culture flasks were coated with 2% agarose in PBS to prevent cell adhesion and subsequently rinsed with DMEM without phenol red. 32 mL of the previously prepared spheroid suspension were then added in each culture flask and incubated at 37 °C, 5% CO2 for 72 h. Following the EV production, the spheroid suspension was collected and centrifuged at 200 g for 3 min to separate spheroids from the EV‐conditioned medium (CM). EV CM was then centrifuged at 2000 g and 4 °C for 10 min before being stored at 4 °C for further use. Spheroid pellets were resuspended in 400 µL of 220‐nm‐filtered DMEM without phenol red and stored at room temperature before further use.

### EV Purification by Size Exclusion Chromatography

Previously collected conditioned media (CM) were concentrated by filtration into 0.5 mL of concentrated conditioned media (CCM) using 100 kDa cut‐off centrifugal filters (Sigma, Centricon Plus‐70 100 kDa, UFC710008) following the supplier instructions. CCM were subsequently purified by size exclusion chromatography. Briefly, SEC columns (Izon Sciences, qEVoriginal/70 nm Gen2 SEC columns, ICO‐70) were rinsed with 25.5 mL of 220nm‐filtered DMEM without phenol red and 0.5 mL of CCM were added to the top of the columns, followed by 220nm‐filtered DMEM without phenol red. The first 2 mL fraction was discarded, and the following 2.4 mL fraction (EV fraction) was collected. EV fractions were then reconcentrated by filtration into 50 to 100 µL of concentrated EV suspension (EV pool) using 10 kDa cut‐off centrifugal filters (Millipore, Amicon Ultra‐4, UFC8010). EV pools were then stored at 4 °C for further analysis or preserved at −80 °C for long‐term storage.

### Cryogenic Transmission Electron Microscopy (cryoTEM)

A 5 µL drop of the initial sample solution was deposited on quantifoil (Quantifoil Micro Tools GmbH) carbon membrane grids. The excess of liquid on the grid was absorbed with a filter paper and the grid was quench‐frozen quickly in liquid ethane to form a thin vitreous ice film using a homemade mechanical cryo plonger. Once placed in a Gatan 626 cryo‐holder cooled with liquid nitrogen, the samples were transferred in the microscope and observed at low temperature (−180 °C). Cryo‐TEM images were recorded on Ultrascan 1000, 2k x 2k pixels CCD camera (Gatan), using a LaB6 JEOL JEM2100 (JEOL, Japan) cryo‐microscope operating at 200 kV with a JEOL low dose system (Minimum Dose System, MDS, JEOL) to protect the thin ice film from any irradiation before imaging and reduce the irradiation during the image capture.

### Particle Quantification and Size Assessment by Nanoparticle Tracking Analysis (NTA)

Samples were centrifuged at 2000 g and 4 °C for 10 min before being diluted between ≈2 × 10^7^ and 2 × 10^8^ particles mL^−1^. NTA was performed on the diluted samples using the NanoSight NS300 (Malvern) along with the NTA 3.4 software. During analysis, the camera level was set at 16, the flow rate was adjusted within the range of 20 of 30 µL min^−1^ and the detection threshold was set at 4.

### Toxilight Toxicity Assay

80 µL of Toxilight reagent (Lonza, LT107‐217) were combined with 20 µL of the previously collected conditioned media in wells of a 96‐well plate (PerkinElmer, Optiplate‐96 F HB, Black) and left at room temperature. Following a 15‐min incubation period, luminescence was measured using a plate reader with a measurement time of 0.1 s. A lysed sample of spheroids with the same spheroid concentration was used as positive control., and 20 µL of DMEM without phenol red with 80 µL of Toxilight reagent was used as blank.

### Live/Dead Viability Assay and Spheroid Integrity Measurement

100 µL of Live/Dead reagent (Invitrogen 488/570) ware combined with 50 µL of the resuspended spheroid pellet in wells of a 96‐well plate (PerkinElmer, Optiplate‐96 F HB, Black) and left at room temperature. Following a 30‐min incubation period, spheroids were imaged using a plate reader both in bright field and in fluorescence mode, with excitation wavelength = 465 nm for Live (green) and 525 nm for Dead (orange). Live and Dead channel were then combined using and a particle analysis was performed using Fiji software to obtain the diameter of each particle. Particles were considered intact spheroids if their diameter was greater to half of the mean spheroid diameter before stimulation, and as damaged spheroids otherwise. Volume of intact and damaged spheroids were estimated with their diameters, and the number of cells per intact or damaged spheroid was estimated by dividing the spheroid volume to an average cell volume. Integrity was finally calculated as the ratio between total number of cells in intact spheroids with the total number of cells.

### Bead‐Based Multiplex Flow Cytometry Assay

EVs were subjected to bead‐based multiplex analysis by flow cytometry (MACSPlex EV Kit IO, human, Miltenyi). Samples were processed according to manufacturer's instructions. Briefly, particle counts quantified by NTA were used to estimate input EV amounts. 5 × 10^8^ EVs were diluted with MACSPlex buffer to a final volume of 120, and 15 µl of MACSPlex Exosome Capture Beads were added. Samples were incubated on an orbital shaker overnight at room temperature protected from light. After washing, samples were incubated with a mix of APC‐conjugated anti‐CD9/CD81/CD63 detection antibodies for 1 h at room temperature. Flow cytometry analysis was performed with Aurora analyzer (Cytek) and data was analyzed with FlowJo software (v10, FlowJo LLC). The 39 single bead populations were gated to allow determination of the APC signal intensity on the respective bead population. Median fluorescence intensity (MFI) for each capture bead was measured and background corrected by dividing MFI values by the signal from matched non‐EV controls that were treated exactly like EV‐containing samples.

### Western Blotting

30 µL of purified extracellular vesicles were resuspended in 4x Laemmli Sample buffer (Bio‐Rad), boiled 10 min at 95 °C and loaded on 4–15% Mini‐Protean TGX Stain‐Free gels (Bio‐Rad), under non‐reducing conditions. Transferred membranes (Immuno‐Blot PVDF Bio‐Rad) were incubated with primary anti‐human antibodies: CD63 (clone H5C6, BD Bioscience 557305 1/1000), CD81 (clone TS81, Medix Biochema 1/1000), SDCBP (syntenin‐1, clone EPR8102, Abcam 1/1000) and 14‐3‐3 (Clone EPR6380, Abcam, 1/1000). Secondary antibodies included HRP‐conjugated goat anti‐rabbit IgG (H + L) (Jackson 111‐035‐144) and HRP‐conjugated goat anti‐mouse IgG (H + L) (Jackson 111‐035‐146). Membranes were developed using Clarity western ECL substrate (Bio‐Rad) and the ChemiDoc Touch imager (Bio‐Rad), with an exposure time of 16.250 s.

### Radioimmunoprecipitation Assay (RIPA) Lysis

EV samples were lysed using RIPA buffer (Thermo Fisher Scientific, 89901) containing 20 mM Tris.HCl pH 7.6, 150 mM NaCL, 1% NP‐40, 1% sodium deoxycholate, and 0.1% SDS. A total of 2.5 µL of EV sample was mixed with 15 µl of RIPA and incubated for 30 min in ice, then sonicated for 30 s with 50% pulse. EV lysates were then diluted by adding 132.5 µL of PBS to achieve a dilution factor of 1:10 in RIPA.

### Protein Quantification Assay

Protein quantification of EV was performed using the Micro BCA protein assay kit (Thermo Fisher Scientific, 23235). A standard curve was prepared using Bovine Serum Albumine (BSA), with final concentrations of 200, 40, 20, 10, 5, 2.5, 1, 0.5 and 0 µg mL^−1^. Protein quantification assays were then conducted as indicated in manufacturer's instructions. Briefly, 45 µL of each sample and of each standard were pipetted in triplicates in wells of a 384‐well microplate (PerkinElmer, Optiplate‐384 F HB, Black). 45 µL of the prepared BSA working reagent were then added to each well, and the plate was placed on a plate shaker for 30 s, before being covered and incubated at 37 °C for 2 h. Absorbance at 562 nm of each well was then measured using a plate reader (PerkinElmer, Ensight Multimode Plate Reader), and protein concentrations were derived from the standard curve.

### LC‐MS/MS Sample Preparation

5 µg protein pellets were dried under vacuum (Savant Centrifuge SpeedVac concentrator, Thermo Fisher Scientific). Dry pellets were solubilized and reduced successively in 10 µL 8 m urea, 100 mm ammonium bicarbonate, 5 mm dithiothreitol, pH 8.0 at 57 °C for 1 h. The protein mixture was then cooled to room temperature followed by the addition of iodoacetamide to 10 mM. The alkylation of the cysteine residues was continued in the dark for 30 min at room temperature. After diluting to 1 m urea with 100 mm ammonium bicarbonate pH 8.0, samples were trypsine/LysC (1 µg, Promega) digested in a total volume of 100 µL by vortexing at 37 °C overnight. Samples were then loaded onto homemade C18 StageTips for desalting. Peptides were eluted using 40/60 CH3CN/H2O + 0.1% formic acid and vacuum concentrated to dryness. Five independent biological replicates of each sample were analyzed.

### LC‐MS/MS Analysis

Liquid chromatography (LC) was performed with a Vanquish Neo LC system (Thermo Scientific) coupled to an Orbitrap Astral mass spectrometer (MS), interfaced by a Nanospray Flex ion source (Thermo Scientific). Peptides were injected onto a C18 column (75 µm inner diameter x 50 cm double nanoViper PepMap Neo, 2 µm, 100Å, Thermo Scientific) regulated at a temperature of 50 °C, and separated with a linear gradient from 100% buffer A (100% H2O + 0,1% formic acid) to 28% buffer B (100% CH3CN + 0,1% formic acid) at a flow rate of 300 nL min^−1^ over 104 min. Peptides were analyzed in the MS applying a 2200 V spray voltage, funnel RF level at 40, and a heated capillary temperature set to 285 °C. MS full scan (380–980 m z^−1^) were recorded in centroid mode using a resolution of 240000 at m/z 200, a normalized AGC target of 500%, and a maximum injection time of 5 ms. The fragment spectra were acquired in DIA mode, with a precursor mass range of 380–980 m z^−1^ with 2 Da isolation windows (without overlap). Isolated precursors were fragmented in the HCD cell using 25% normalized collision energy, a normalized AGC target of 500%, and a maximum injection time of 3 ms.

### LC‐MS/MS Data Processing

For identification, the data were searched against the Homo sapiens (UP000005640) Uniprot database using Pulsar search engine through Spectronaut v19 (Biognosys) by directDIA+ analysis using default search settings. Enzyme specificity was set to trypsin and a maximum of two missed cleavage sites was allowed. Carbamidomethyl was set as a fixed modification and N‐terminal acetylation and oxidation of methionine as variable modifications. The resulting files were further processed using myProMS v3.10, https://github.com/bioinfo‐pf‐curie/myproms.^[^ ^76^ ^]^

For protein quantification, ion XICs (sum of fragment peak areas) from proteotypic peptides shared between compared conditions (TopN matching) were used, with missed cleavages and carbamidomethylation allowed. Median and scale normalization at peptide level was applied on the total signal to correct the XICs for each biological replicate (N = 5). To evaluate the statistical significance of the change in protein abundance, a linear model (adjusted on peptides and biological replicates) was performed, and a two‐sided *T*‐test was applied on the fold change estimated by the model. The p‐values were then adjusted using the Benjamini–Hochberg FDR procedure. LFQ quantification was also performed following the algorithm, as described,^[^ ^77^ ^]^ with the minimum number of peptide ratios set to 2 and the large ratios stabilization feature.

Proteins were selected with at least 2 distinct peptides across 5 biological replicates of an experimental condition and that showed a log2(fold change) ≥ 2 or ≤ −2 and an adjusted p‐value of less than 0.05. Proteins with a log2(fold change) between −2 and 2 were considered as common to two conditions. The mass spectrometry proteomics raw data have been deposited to the ProteomeXchange Consortium via the PRIDE partner repository^[^ ^78^ ^]^ with the dataset identifier PXD054065 (Username: reviewer_pxd054065@ebi.ac.uk; Password: BKcMFvyR6Ux1).

### Wound Healing Assay

Human skin fibroblasts (HSFs) were seeded in 96‐well plates (TPP, 92096) at a density of 100 000 cells per well, and cultured at 37 °C, 5% CO2 for 5 to 7 days until confluency. A scratch wound was then made using a 3D printed guide and a sterile 100 µL pipette tip and the medium was replaced with fresh serum‐free culture medium, fresh serum‐free culture media supplemented with 4% and 10% FBS, fresh serum‐free culture medium supplemented with EVs produced by starvation (10^9^ EVs mL^−1^, either 2D or 3D), fresh serum‐free culture medium supplemented with EVs produced in the cross‐slot chip at Re = 208, Re = 313 and Re = 417 (10^9^ EVs mL^−1^). Cells were then incubated for 96 h at 37 °C; 5% CO2 and regularly imaged using a plate reader (PerkinElmer, Ensight Multimode Plate Reader). At each time point, total area covered by cells in the scratch wound was measured using Fiji software. Area coverage was then calculated as the area covered by the cells in the scratch wound divided by the total area of the wound.

### Neo‐Angiogenesis Assay

30 µL of a 2 mg mL^−1^ collagen I solution () previously prepared in ice was added in wells of a 384‐well plate After a 30‐min incubation period at 37 °C, 50 µL of a HUVEC spheroids suspension at ≈100 spheroids mL^−1^ were added onto the polymerized collagen. Plates were left at room temperature for 20 min to allow spheroid sedimentation, and culture media were replaced by either 50 µL of VEGF‐α (Sigma, 01–185) (12.5, 25 and 50 ng mL^−1^), 50 µL of EV pool produced in cross‐slot chips diluted in DMEM (5 × 10^9^ EVs mL^−1^), 50 µL of EV pool produced through starvation diluted in DMEM (5 × 10^9^ EVs mL^−1^) or 50 µL of DMEM only. Wells were then imaged in bright‐field microscopy after 24 h of incubation. The sprout length and the number of sprouts per spheroid were then quantified using FiJi software.

### Statistical Analysis

If applicable, statistical differences between groups were evaluated using an unpaired, two‐tailed Student's *t*‐test. *P*‐values of *p* > 0.05, *p* < 0.05, *p* < 0.01 and *p* < 0.001 were indicated as ‐, ^*^, ^**^ and ^***^, respectively. All statistical analyses were conducted using GraphPad Prism software (v 9.3.1).

## Conflict of Interest

The authors declare no conflict of interest.

## Supporting information



Supporting Information

Supplemental Movie 1

Supplemental Movie 2

## Data Availability

The data that support the findings of this study are available from the corresponding author upon reasonable request.
