# Supplementary material for: High‐Yield Bioproduction of Extracellular Vesicles from Stem Cell Spheroids via Millifluidic Vortex Transport
Source: Adv Mater. 2024 Nov 12;37(20):2412498. doi: 10.1002/adma.202412498 (PMC12087746; doi:10.1002/adma.202412498)
Supplement: Supplementary file 1 — Supporting Information [file ADMA-37-2412498-s002.pdf]

ADVANCED MATERIALS

Supporting Information

for *Adv. Mater.*, DOI 10.1002/adma.202412498

High-Yield Bioproduction of Extracellular Vesicles from Stem Cell Spheroids via Millifluidic Vortex Transport

*Elliot Thouvenot, Laura Charnay, Noa Burshtein, Jean-Michel Guigner, Léonie Dec, Damarys Loew, Amanda K.A. Silva, Anke Lindner and Claire Wilhelm**

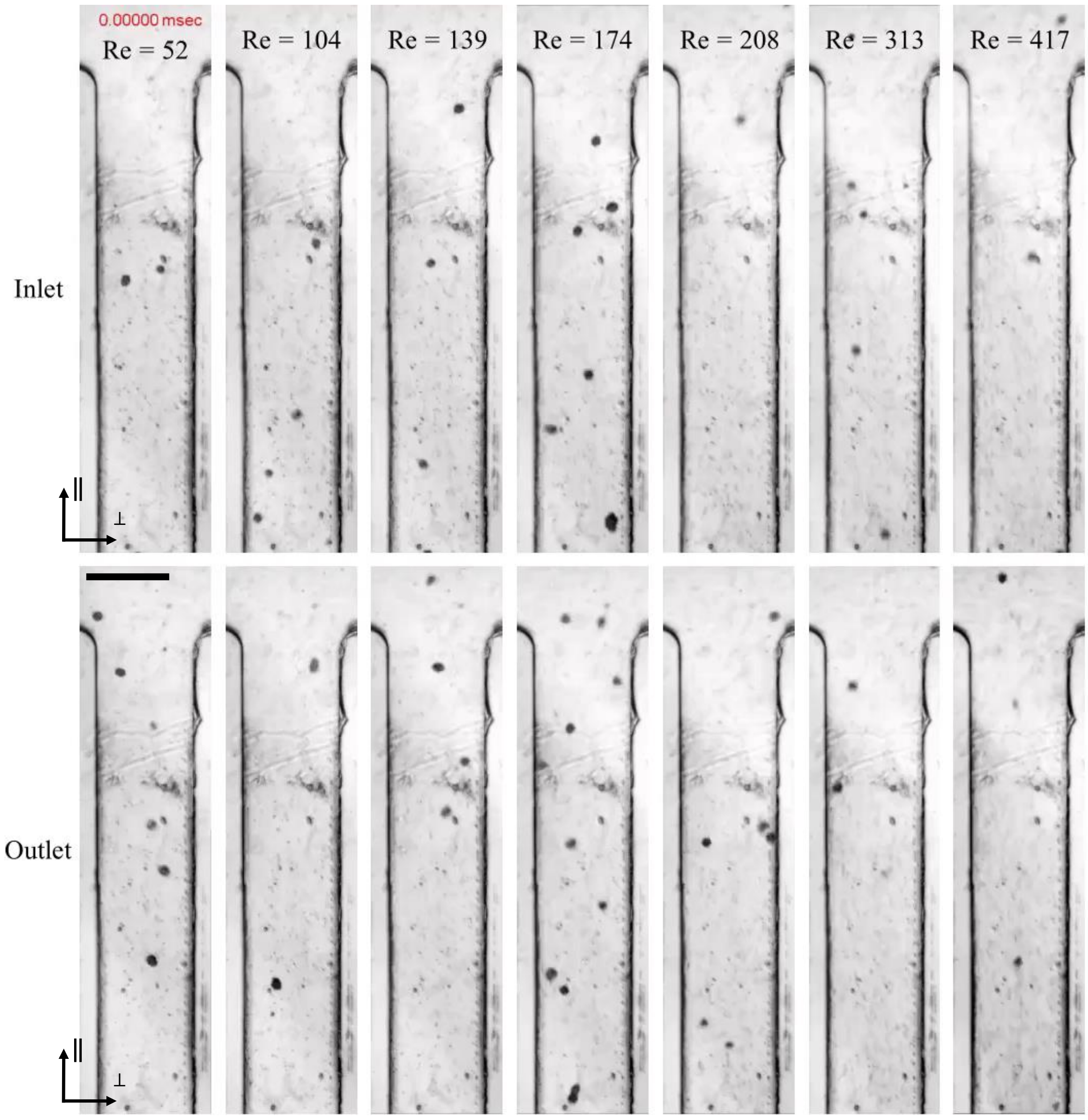

Supplementary Movie 1. Video recording depicting the spheroids transported within the inlet and outlet channels at various Reynolds numbers. Scale bar = 1 mm, magnification = 2.5X.

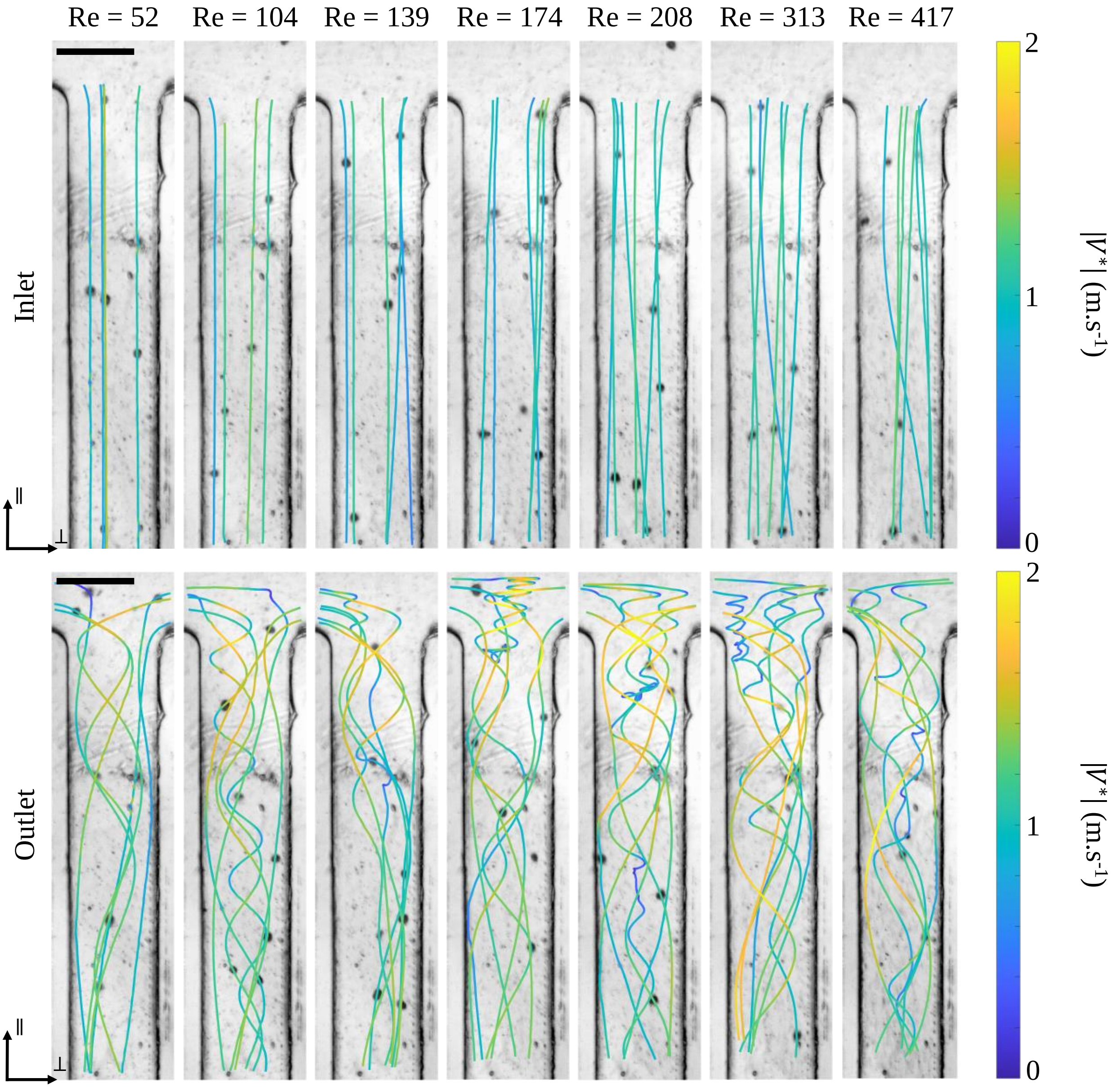

Figure S1. Snapshots extracted from tracking movies showing spheroid trajectories within the inlet and outlet channels. Trajectories are color-coded based on the normalized velocity V*, calculated as the ratio between the magnitude of the projected velocity of the spheroid in the plane x-y and the average velocity of the fluid. Scale bars = 1 mm, magnification = 2.5X.

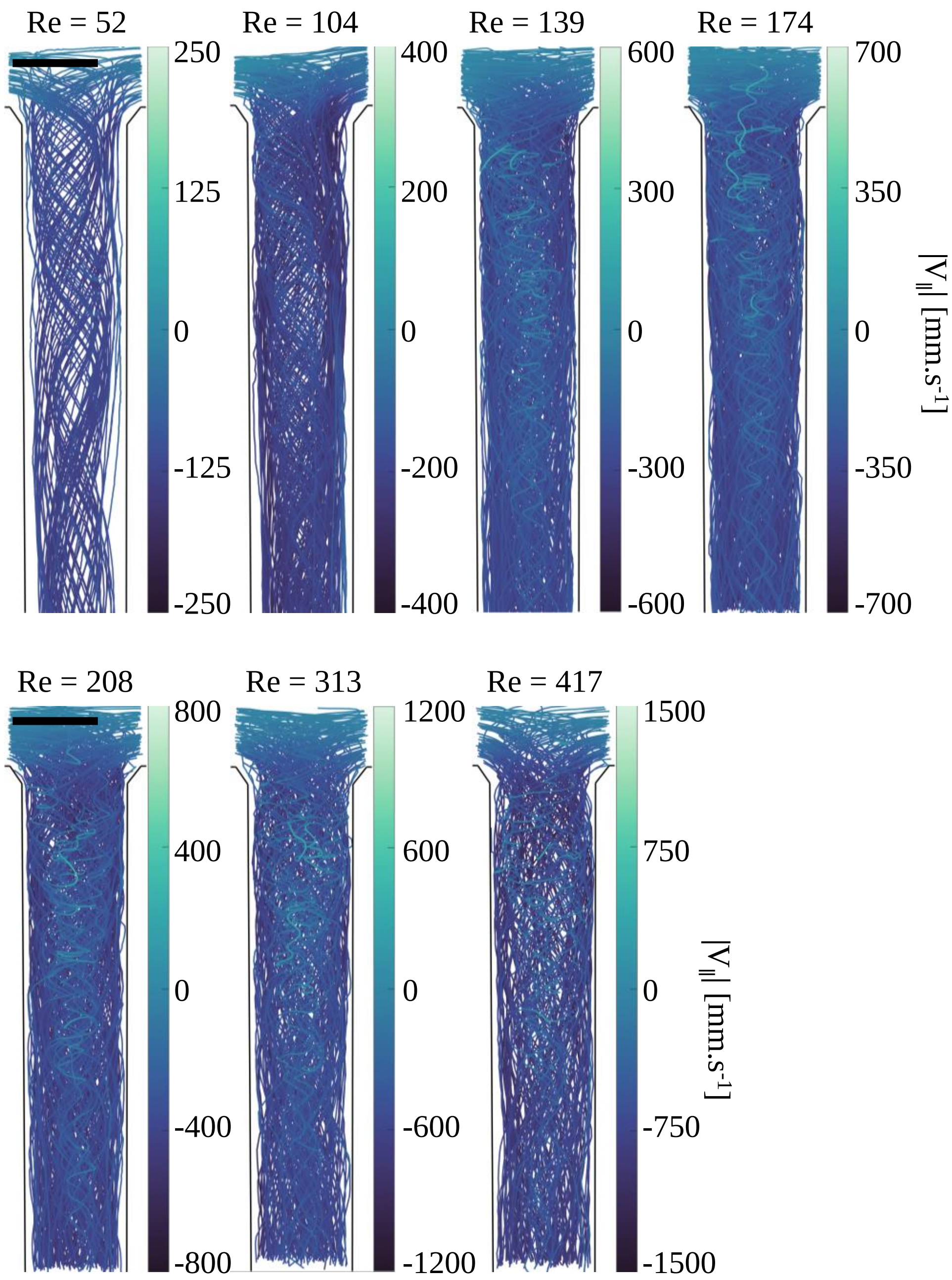

Figure S2. Spheroid trajectories within the outlet channels for various Reynolds numbers color-coded based on the magnitude of the longitudinal velocity projected in the x-y plane, in millimeters per second. Scale bars = 1 mm.

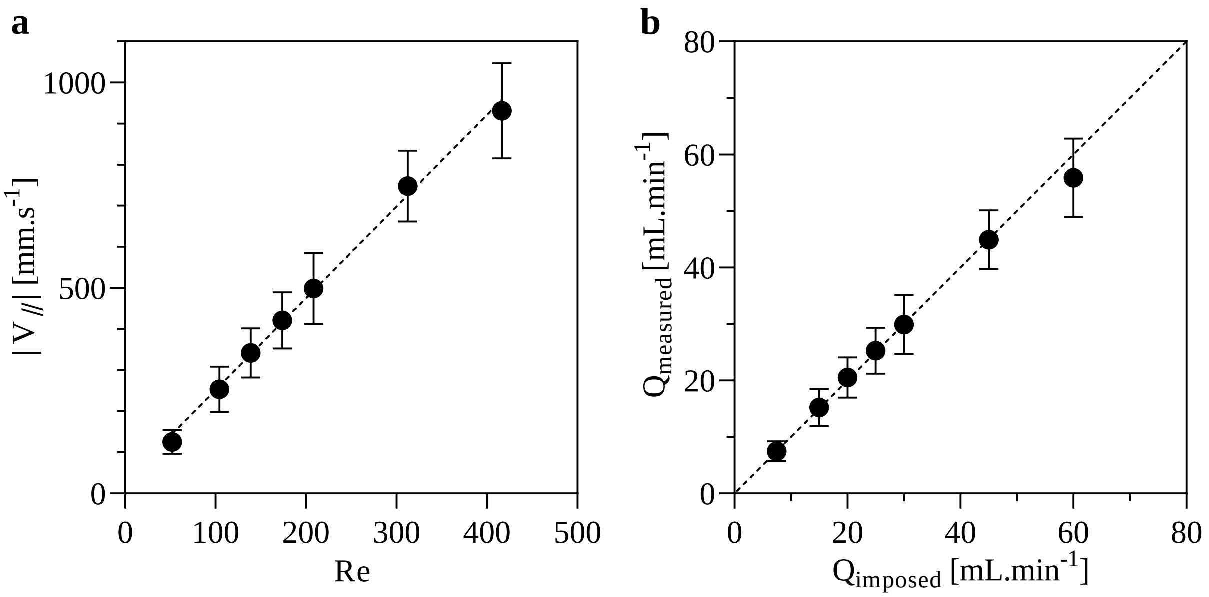

Figure S3. Longitudinal velocity and flow rate in the inlet channel. (a) Average magnitude of the mean longitudinal velocities in the inlet channel along each trajectory plotted against Reynolds number. Results are presented as mean ± SD (n = 80 to 216, depending on the condition). Dotted lines indicate linear fit. **(b)** Flow rates calculated from average of the mean longitudinal velocities within the inlet channel plotted against the imposed flow rates. Results are presented as mean ± SD (n = 80 to 216, depending on the condition). The dotted line indicates the identity line.

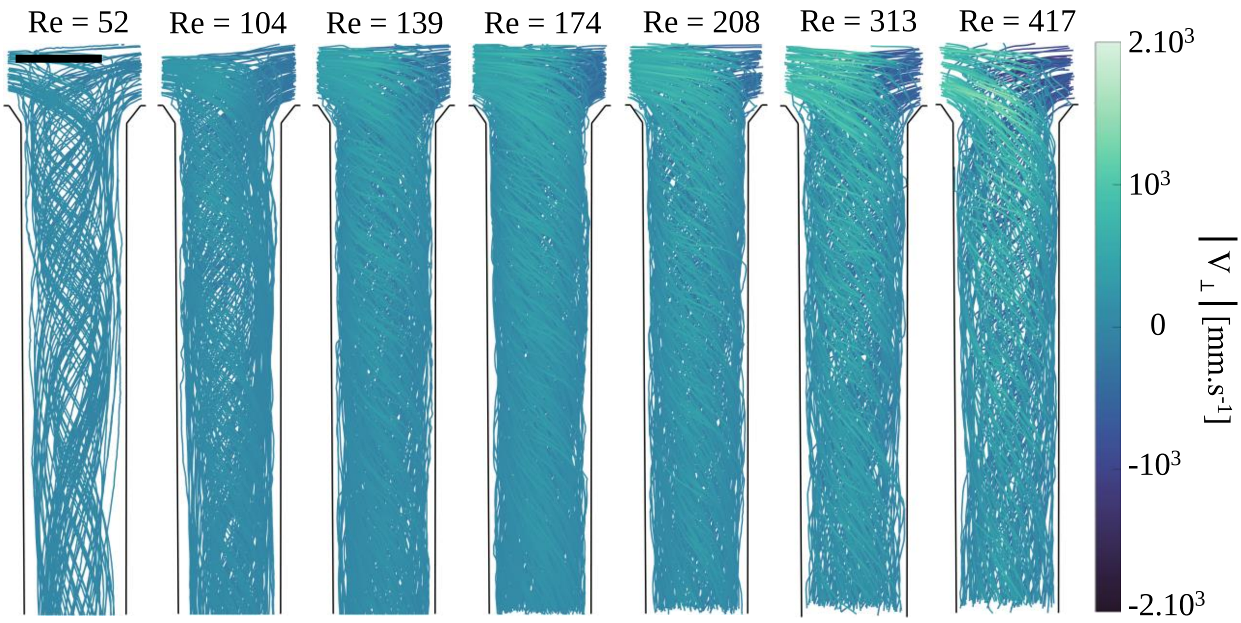

Figure S4. Spheroid trajectories within the outlet channels for various Reynolds numbers color-coded based on the magnitude of the orthogonal velocity projected in the x-y plane, in millimeters per second. Scale bars = 1 mm.

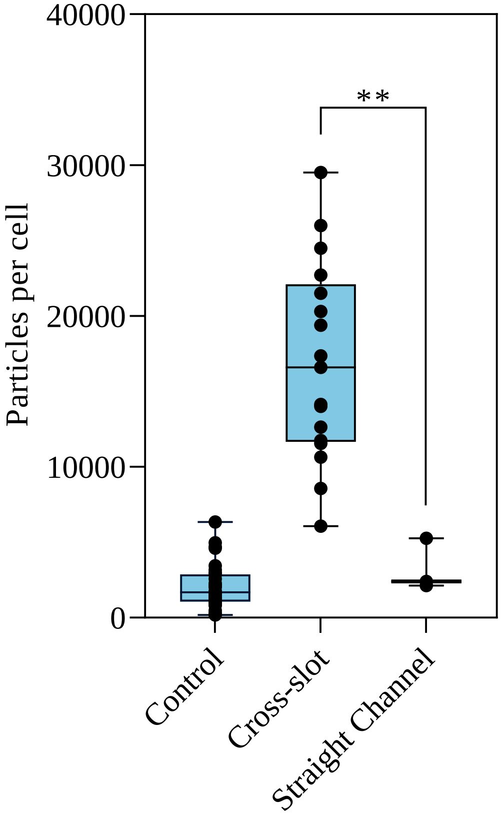

Figure S5. Quantification of particles produced per cell by unstimulated hMSC spheroids (Control), hMSC spheroids stimulated in the cross-slot device (Cross-Slot) and hMSC spheroids stimulated in a straight channel (Straight Channel). Each data point represents an independant experiment. ** = p < 0,01 (Student t-test).

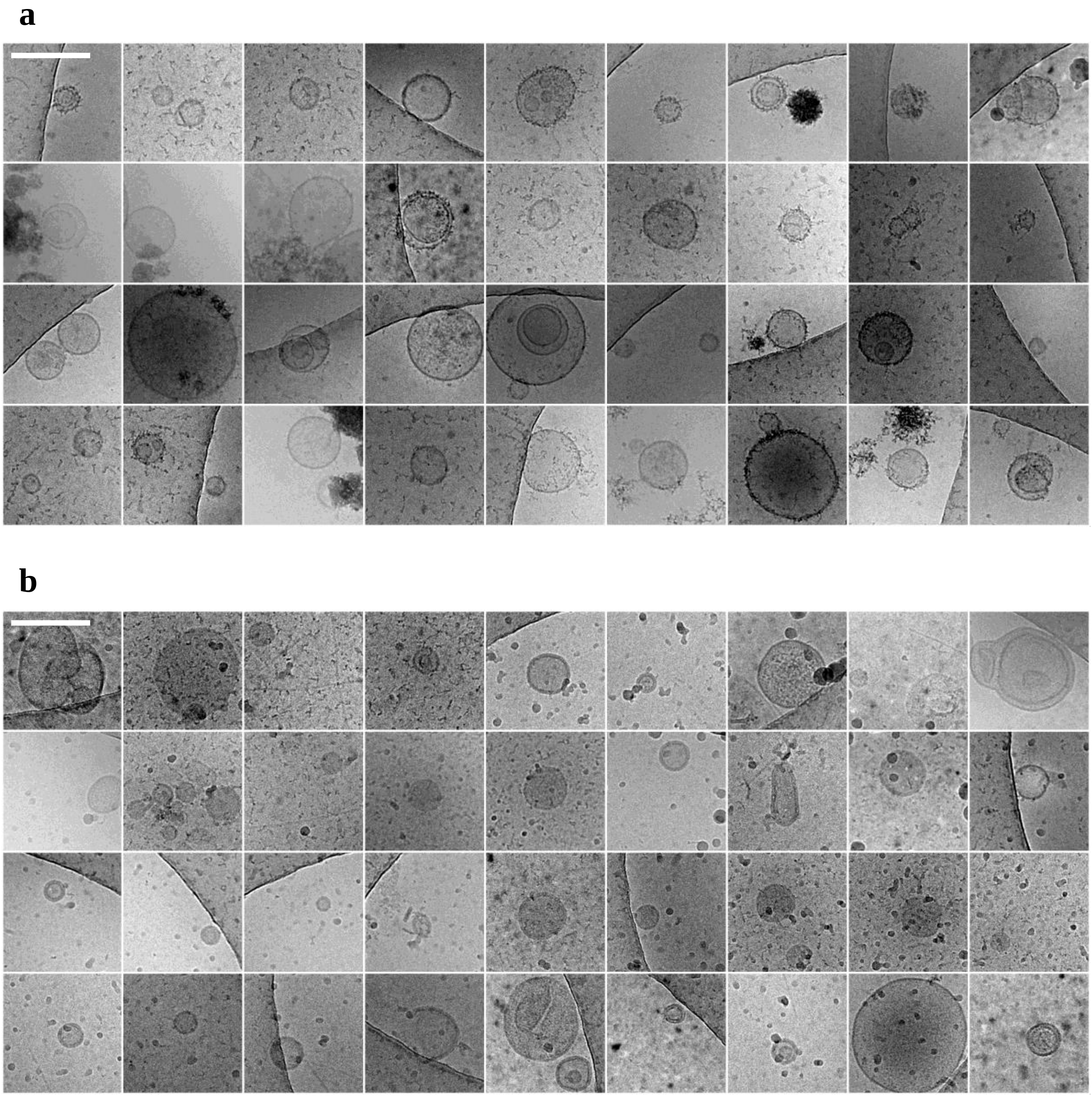
a
b

c

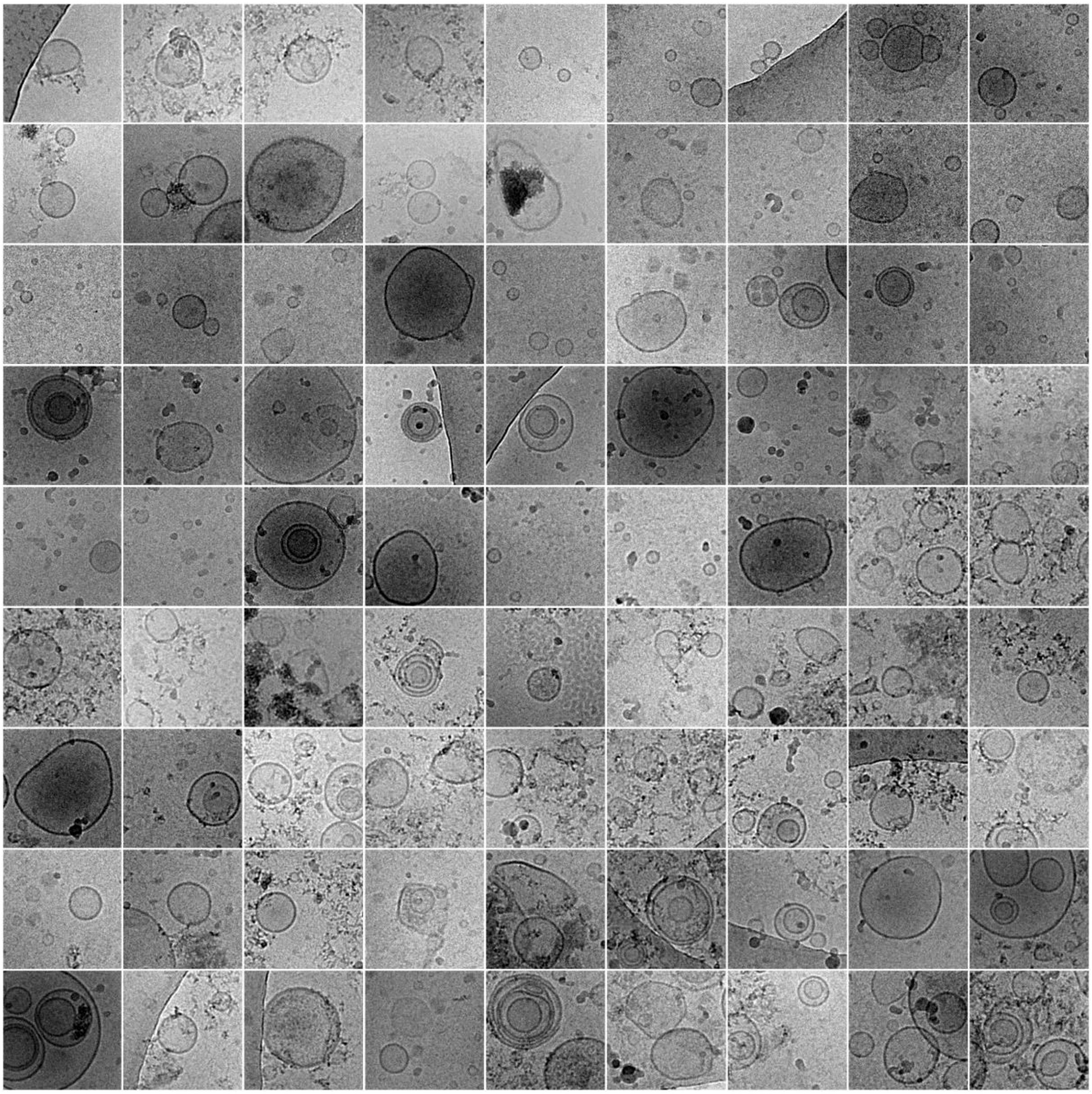

d

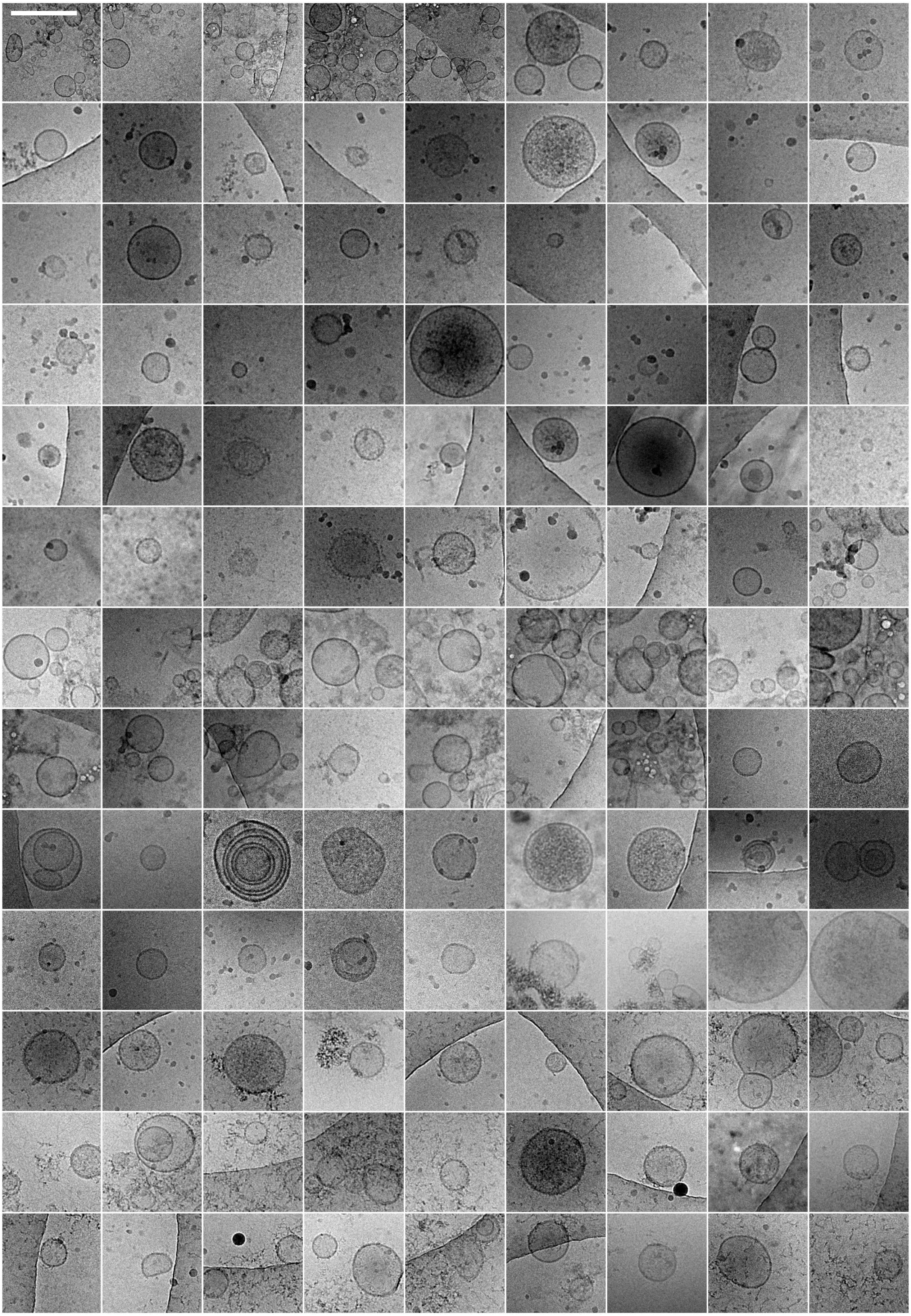

e

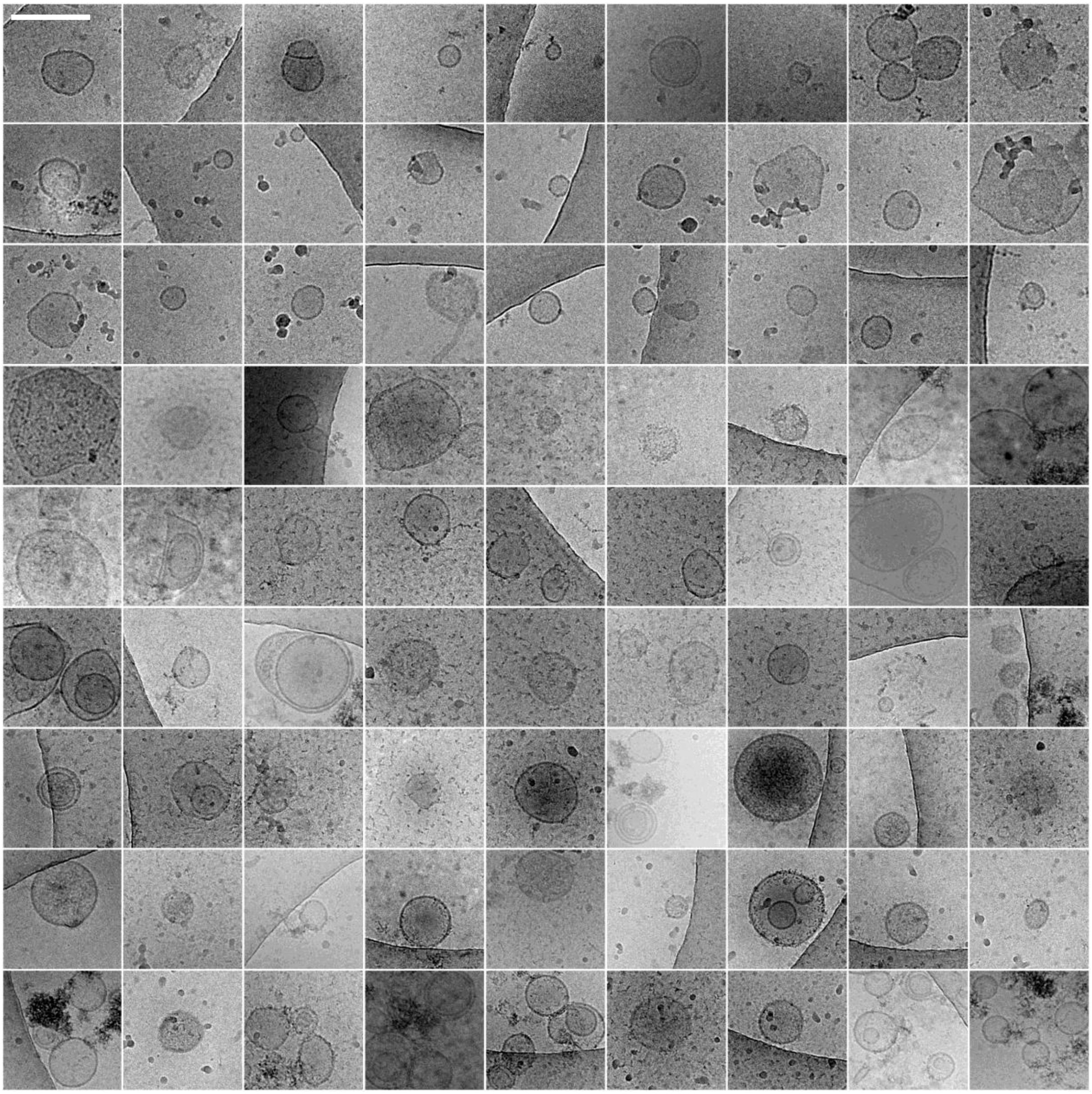

Figure S6. Cryogenic transmission electron microscopy of extracellular vesicles produced from **(a)** serum-deprived hMSC monolayers, **(b)** serum-deprived hMSC spheroids and hMSC spheroids stimulated in the cross-slot chip at **(c)** Re = 208, **(d)** Re = 313 and **(e)** Re = 417. Scale bar = 200 nm.

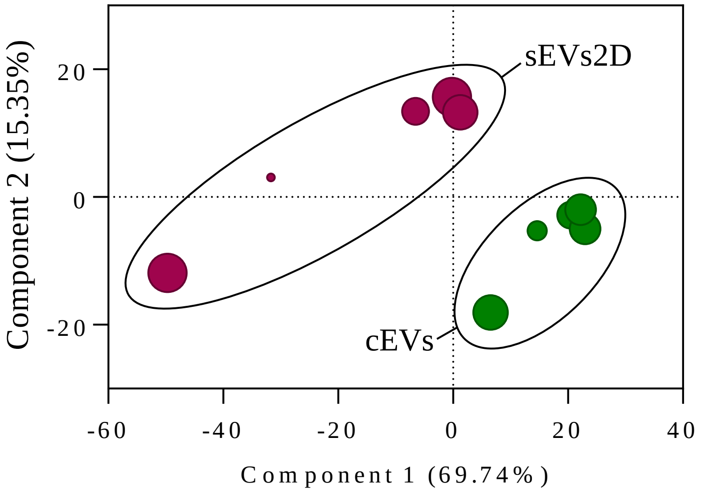

Figure S7. Principal component analysis of EVs produced from hMSC in 2D configuration through serum starvation (sEVs 2D, red circles) and EVs produced from hMSC spheroids stimulated in the cross-slot chip (cEVs, Re = 313, green circles). The size of the circles represents the third component (6.5%). Each circle represents an independent biological replicate.

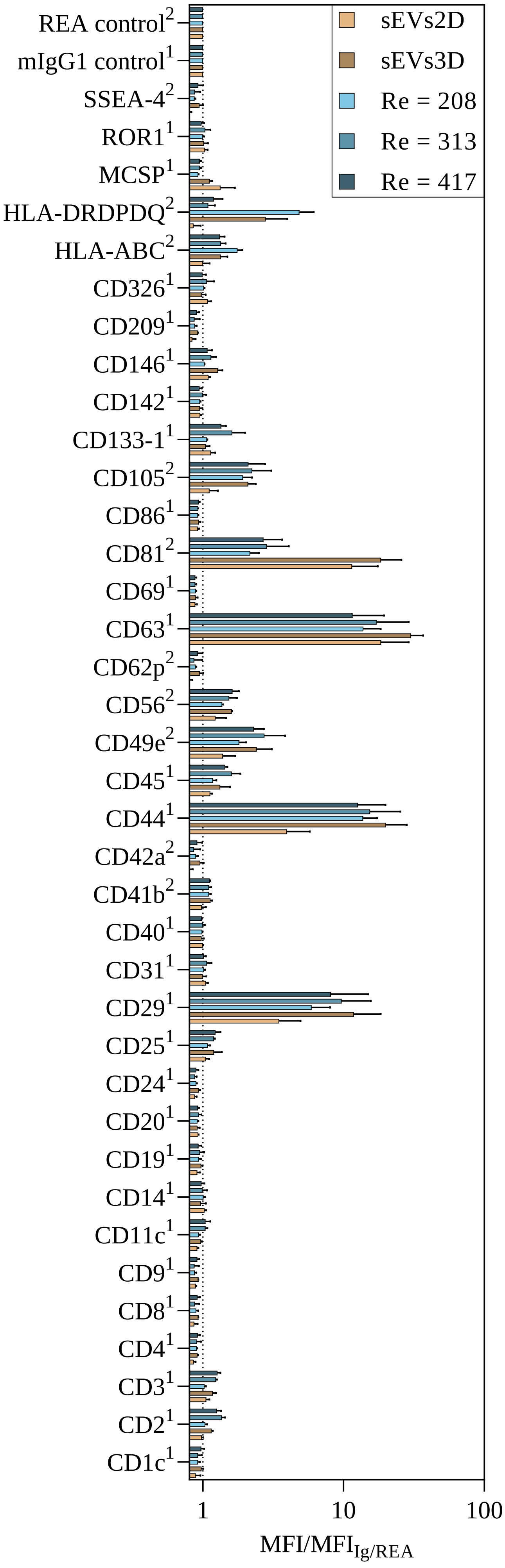

Figure S8. Bead-based fluorescence flow cytometric analysis of extracellular vesicles produced through serum starvation from hMSC in a 2D configuration (sEVs2D) or from hMSC spheroids (sEVs3D), or from hMSC spheroids stimulated in the cross-slot device at Re = 208, Re = 313 and Re = 417. Results are presented as ratios of mean fluorescence intensity with respect to mIgG1 (1) or REA (2) controls. Results are shown as mean ± SD (n = 3).

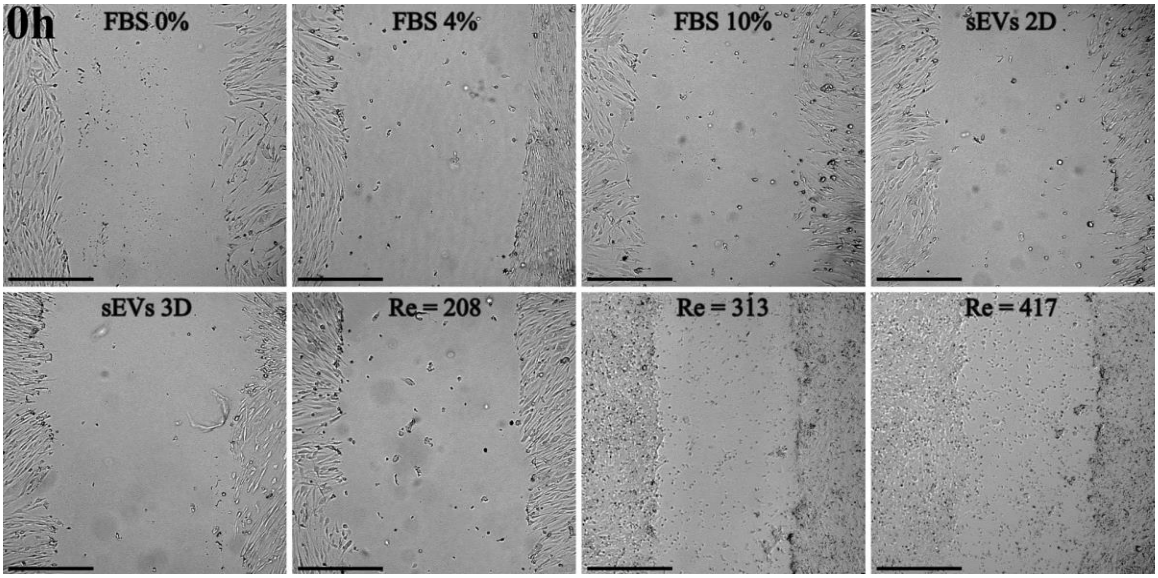

Supplementary Movie 2. Video recording depicting the wound closure over time for cells incubated in **(a)** serum-deprived culture medium (FBS 0%), **(b)** culture medium supplemented with 4% FBS (4% FBS), **(c)** culture medium supplemented with 10% FBS (10% FBS), **(d)** culture medium supplemented with EVs produced through serum starvation from hMSC in 2D configuration (sEVs 2D), **(e)** culture medium supplemented with EVs produced through serum starvation from hMSC spheroids (sEVs 3D) and **(f)** culture medium supplemented with EVs produced from hMSC spheroids stimulated in the cross-slot device (cEVs). Scale bar = 150 µm.

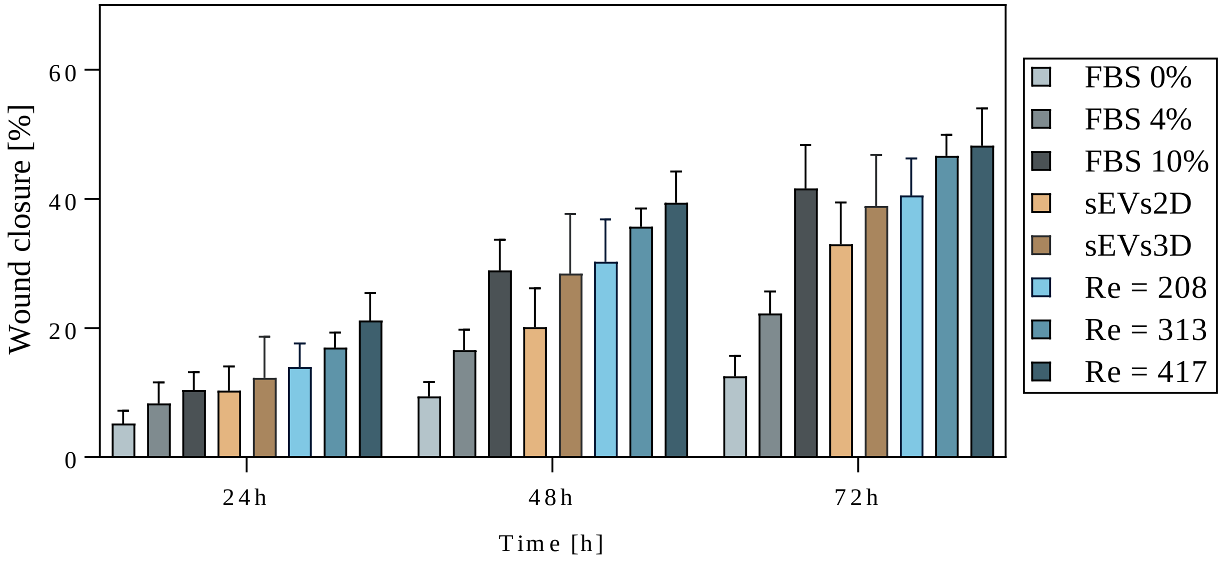

Figure S9. Wound closure after 24, 48 and 72 hours of incubation, calculated as the area covered by human skin fibroblasts in the wound, for cells incubated in serum-deprived culture medium (FBS 0%) or culture medium supplemented with 4% or 10% FBS, EVs produced through serum starvation from hMSC in 2D configuration (sEVs2D) or hMSC spheroids (sEVs3D) and EVs produced from hMSC spheroids stimulated in the cross-slot chip at Re = 208, Re = 313 or Re = 417. Results are shown as mean ± SD (n = 6 – 15 depending on the condition)

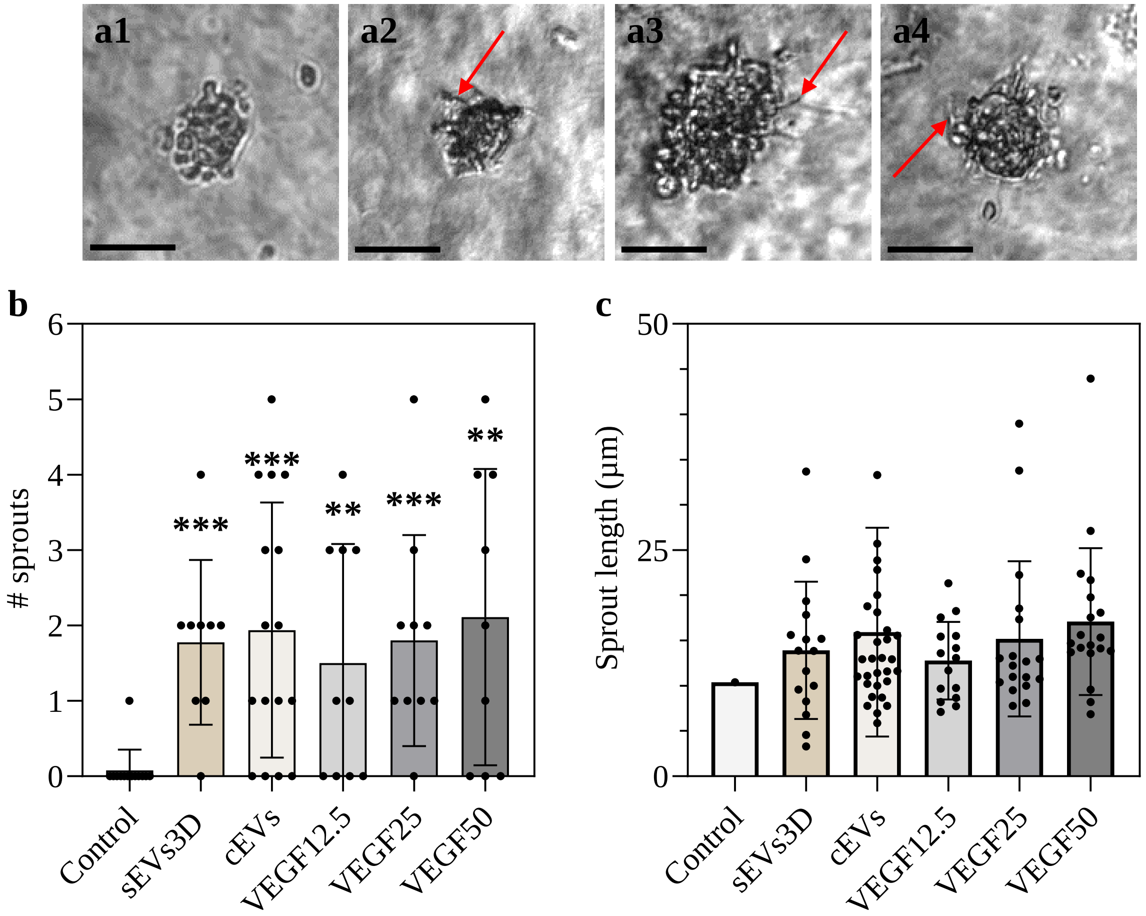

Figure S10. Enhanced angiogenesis properties. (a) Bright-field images of HUVEC spheroids after 24 incubation in **(a1)** serum-deprived culture medium or culture medium supplemented with **(a2)** EVs produced through starvation from hMSC spheroids, **(a3)** EVs produced through from hMSC spheroids stimulated in the cross-slot device at Re = 313 and **(a4)** 25 ng/mL of VEGF-α. Red arrows show examples of sprouts. Scale bars = 100 µm. (**b)** Quantification of sprouts on HUVEC spheroids embedded in collagen after 24 hours of incubation in serum-deprived culture medium (Control) or culture medium supplemented with EVs produced through starvation from hMSC spheroids (sEVs3D), EVs produced from hMSC spheroids stimulated in the cross-slot device at Re = 313 (cEVs) or 12.5 ng/mL, 25 ng/mL and 50 ng/mL of VEGF-α. Results are presented as mean ± SD. Each data point represents a single HUVEC spheroid. Stars denote the statistical significance of the difference with the control group (** = $p < 0.01$ and *** = $p < 0.001$, Student t-test) **(c)** Quantification of sprout lengths on HUVEC spheroids embedded in collagen after 24 hours of incubation in serum-deprived culture medium (Control) or culture medium supplemented with EVs produced through starvation from hMSC spheroids (sEVs 3D), EVs produced from hMSC spheroids stimulated in the cross-slot device (cEVs) or 12.5 ng/mL, 25 ng/mL and 50 ng/mL of VEGF-α. Results are presented as mean ± SD. Each data point represent a single sprout.
